# A Review of Cerebral Hemodynamics During Sleep Using Near-Infrared Spectroscopy

**DOI:** 10.3389/fneur.2020.524009

**Published:** 2020-11-19

**Authors:** Haoran Ren, Xinyu Jiang, Ke Xu, Chen Chen, Yafei Yuan, Chenyun Dai, Wei Chen

**Affiliations:** ^1^The Center for Intelligent Medical Electronics, School of Information Science and Technology, Fudan University, Shanghai, China; ^2^Human Phenome Institute, Fudan University, Shanghai, China; ^3^Shanghai Key Laboratory of Medical Imaging Computing and Computer Assisted Intervention, Shanghai, China

**Keywords:** cerebral hemodynamics, near-infrared spectroscopy, sleep stages, sleep disorders, cerebrovascular autonomic regulation

## Abstract

Investigating cerebral hemodynamic changes during regular sleep cycles and sleep disorders is fundamental to understanding the nature of physiological and pathological mechanisms in the regulation of cerebral oxygenation during sleep. Although sleep neuroimaging methods have been studied and have been well-reviewed, they have limitations in terms of technique and experimental design. Neurologists are convinced that Near-infrared spectroscopy (NIRS) provides essential information and can be used to assist the assessment of cerebral hemodynamics, and numerous studies regarding sleep have been carried out based on NIRS. Thus, a brief historical overview of the sleep studies using NIRS will be helpful for the biomedical students, academicians, and engineers to better understand NIRS from various perspectives. In this study, the existing literature on sleep studies is reviewed, and an overview of the NIRS applications is synthesized and provided. The paper first reviews the application scenarios, as well as the patterns of fluctuation of NIRS, which includes the investigation in regular sleep and sleep-disordered breathing. Various factors such as different sleep stages, populations, and degrees of severity were considered. Furthermore, the experimental design and signal processing, as well as the regulation mechanisms involved in regular and pathological sleep, are investigated and discussed. The strengths and weaknesses of the existing NIRS applications are addressed and presented, which can direct further NIRS analysis and utilization.

## Introduction

The brain has a complex cerebral autoregulation mechanism which guarantees the normal function during daily activity and nocturnal sleep. The mutual regulation of the neurons, glial cells, and the vasculature of the brain constantly regulate the homeostasis to maintain health. Sleep, as an essential part of physiological activity, facilitates the recovery of humans' physical function in daily life. Humans spend roughly one-third of their lives asleep (1). However, an increasing number of people are undergoing sleep disorders resulting from the accelerated pace of life and the pressure of work. Sleep disorders are associated with several morbidities as well as increased mortality (2), affecting the functional output of the brain in terms of alertness, cognition, and mood (3), as well as increasing the risk of cerebrovascular disease (4).

Polysomnography (PSG), as a gold standard diagnostic test for sleep disorders, provides multiple physiological signals containing brain activity (electroencephalography: EEG), eye movement (electro-oculogram: EOG), chin muscle tone (electromyography: EMG), and oxygen saturation (peripheral capillary oxygen saturation: SpO_2_). The amplitudes and/or frequencies of these physiological signals would change with sleep events. For instance, a specific of EEG signal rhythmicity would be present in specific sleep stages, and the decline of SpO_2_ would resulted from the termination of respiration during apnea. Even though EEG signals during sleep provided significant information for clinical diagnosis and basic researches, the cerebral low frequency oscillations in terms of cerebral hemodynamics could provide supplementary information for a better understanding of sleep neurophysiology which cannot be detected by the EEG (5–8). Moreover, the high energy requirements of the brain renders it more susceptible to hypoxic conditions (9). The detection of arterial oxygen saturation may not provide sufficient information for instantaneous detection of apneic episodes and its effects on brain tissues. Accordingly, investigating cerebral hemodynamic changes during sleep contributes to the understanding of cerebrovascular regulation mechanisms in pathological conditions associated with sleep disorders, such as sleep apnea, and insomnia. Moreover, understanding the similarities and differences between physiological and pathological mechanisms can enhance the efficiency of the treatment and reduce the complications of these common sleep disorders.

Near-infrared spectroscopy (NIRS) as an emerging non-invasive functional imaging technique has the capability to detect the changes of oxygen hemoglobin (HbO_2_), de-oxygen hemoglobin (HHb), and total oxygen hemoglobin (tHb) concentrations. The low-frequency changes in HbO_2_, HHb, and tHb could reflect the brain functional activity involving the cerebral blood supply and cerebral oxygen consumption, as well as sufficient or impaired cerebrovascular autonomic regulation. The synchronous acquisition of EEG and NIRS provides an opportunity to comprehensively understand brain activities in terms of neurovascular regulation.

Based on the advantages of NIRS in detecting cerebral oxygenation, this review focuses on the patterns of cerebral hemodynamic changes during regular sleep cycles and the pathological conditions in patients with sleep-disordered breathing. Even though the experimental studies in patients with sleep-disordered are significant in further understanding of the role of persistent or intermittent apnea in cardiovascular and cerebrovascular injury, as well as cognitive disorders, the related experimental design studies such as cognitive performance and exercise tolerance for patients with sleep-disordered breathing are beyond the scope of this article.

This review is structured as follows: the first section presents the background of the related studies, including the physical principles of NIRS and the potential application of NIRS in sleep studies. The second section is dedicated to discuss the hemodynamic signal changes during sleep state transitions and episodic apnea/hypopnea. Then, the interpretation of physiological and pathological mechanisms mediating sleep transitions and apnea syndromes are introduced. Finally, the last section is devoted to reporting the differences in the experimental design and the signal processing methods. A future perspectives section ends the review.

## Background

### Basic Principles of NIRS

NIRS is an emerging non-invasive functional imaging technique. It progressively gains attention owing to its relatively high temporal resolution (ms), portability, non-invasiveness, low cost, and relative insensitivity to subject movements. It has been widely used in areas involving brain-computer interface (BCI), language, motor task behaviors, psychology, cognition, etc. Details regarding the application of NIRS have been well-reviewed previously (10–12). The technique of NIRS is based on the principles that human tissues are relatively transparent in the near-infrared wavelength range (650–1,000 nm), since the main transmission path is scattering (13). Furthermore, human tissues have particular absorption properties that the domain absorbers, HbO_2_ and HHb located in small vessels (<1 mm diameter), have varying absorption spectra (13, 14). The scattering property in human tissues is typically 100 times larger than absorption (15). NIRS relies on the procedure that the near-infrared light penetrates human tissues and then the detectors obtain the attenuated light emerging from the head. Due to the complex scattering caused by the multi-tissue layers, the optical path-length passing through the tissue is longer than the distance between the source and the detector located on the surface of the scalp. The spatial distribution of the optical path-length exhibits banana-shaped. According to the different illuminating types, there are three different NIRS techniques: continuous wave (CW), frequency-domain (FD), and time-domain (TD) based system. CW-based NIRS system is widely used due to the low cost and portable properties. More details about the differences of the three types of instruments were discussed in the related articles (10, 11). The following NIRS description is mainly focused on the CW-based system.

**Figure 1:**
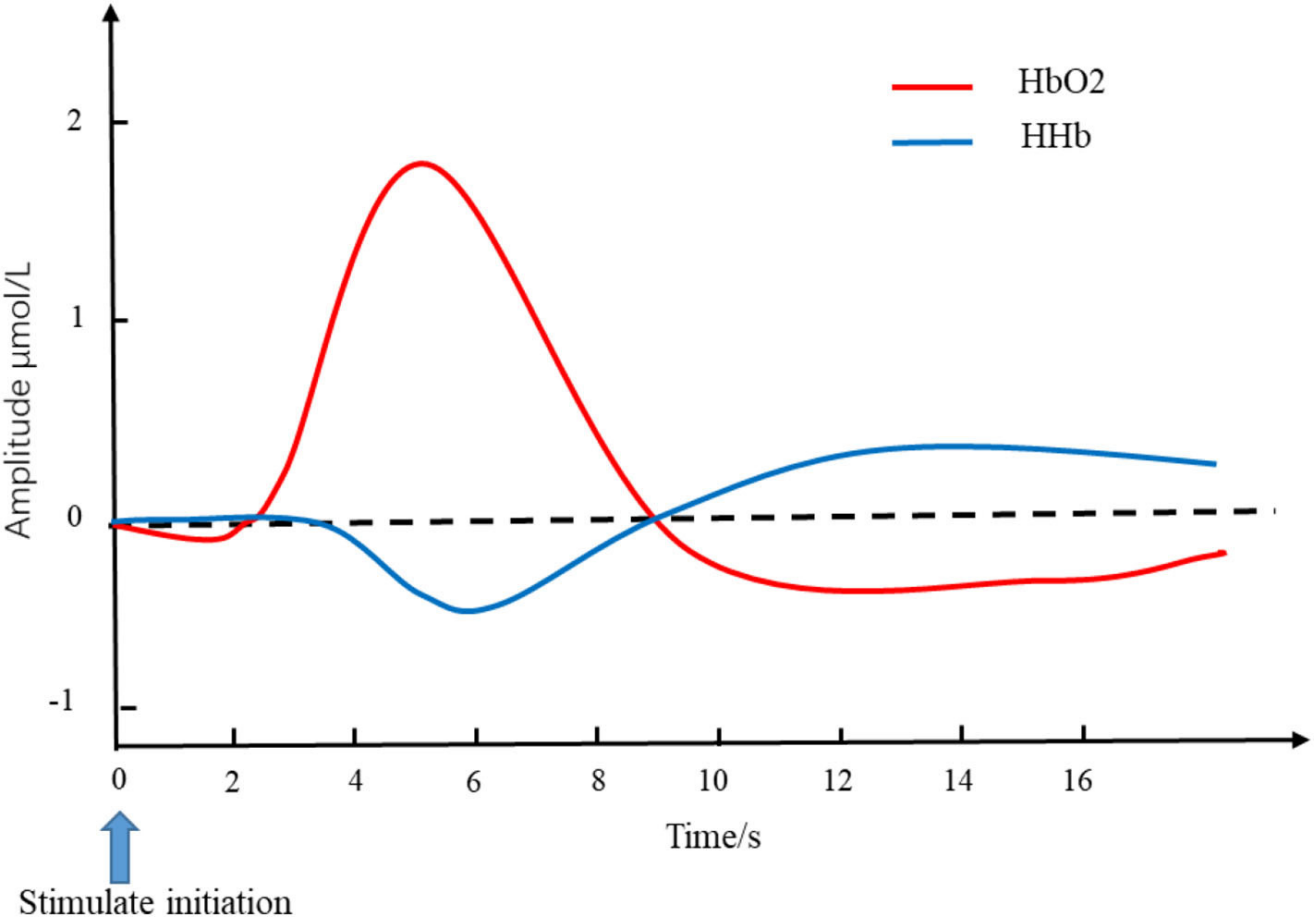
Simulated hemodynamics in response to stimulation. The red line and blue line represent the changes of HbO_2_ and HHb, respectively. The blue arrow pointing at time 0 is the initiation of stimulation. The typical changes of cerebral hemodynamic in response to the external stimulus is an increased HbO_2_ accompanied by a decreased HHb. HbO_2_, oxygen hemoglobin concentration; HHb, de-oxygen hemoglobin concentration.

The attenuated light collected by detectors was converted into the changes in HbO_2_ and HHb by the modified Beer-Lambert law (MBLL). The typical changes in the cerebral hemodynamic response to the external stimulus or cognitive tasks underlying neurovascular coupling mechanism is an increased HbO_2_ accompanied by a decreased HHb (10). The canonical pattern of the hemodynamic response to stimulation is shown in Figure 1. The changes in HbO_2_ and HHb reflect brain activity during different physiological/pathological states or under specific brain tasks (16). A simultaneous increase in HbO_2_ and a stable level or decrease in HHb indicate that the rise of cerebral blood flow (CBF) is larger than cerebral oxygen metabolism (17, 18). The total hemoglobin concentration (tHb), that is, the sum of HbO_2_ and HHb, reflects cerebral blood volume (CBV). Thehemoglobin difference (HbD)—the difference between HbO_2_ and HHb—could reflect the intravascular oxygenation and cerebral blood flow (19).

Excluding the straight forward assessment of cerebral hemoglobin concentrations and derivatives, two indices of cerebral oximetry, which are cerebral tissue oxygenation index (TOI) and regional cerebral oxygen saturation (StO_2_), could provide absolute values of the regional cerebral oxygen saturation. The two parameters were obtained from spatially resolved spectroscopy with multi-distance optodes (20). TOI showed an absolute value of tissue oxygenation in percentage, which was measured at three close distances using the diffusion equation, reflecting cerebral oxygenation, and cerebral venous saturation (21). StO_2_ can be obtained from two interoptode distances with a short distance and a larger distance, reflecting the amount of regional oxyhemoglobin in the unit volume of tissue. Although the two parameters are obtained from two distinct devices and algorithms, the values between the two parameters are comparable and highly correlated (22, 23). The two indices of cerebral oximetry are more concerned in the clinical use.

Changes in cerebral hemodynamics are associated with the autoregulatory system, spontaneous brain action, and systemic physiological components (24). In general, the dynamic changes in HbO_2_ and HHb in human cerebral tissues are contaminated by natural oscillations of cardiac activity at frequencies of 0.6–2 Hz and respiration at frequencies of 0.15–0.6 Hz (25–28). The low frequency oscillations (LFOs)ranging between 0.06 and 0.10 Hz and very low frequency (VLFOs) ranging between 0.01 and 0.05 Hz are important features of cerebral hemodynamics and play an important role in the resting-state and activation-state connective networks of human brain (29, 30). Additionally, spontaneous low and very low hemodynamic oscillations (3–150 mHz) measured with NIRS reflect the endothelial (5–20 mHz), neurogenic (20–50 mHz), and myogenic (50–150 mHz) components of vasomotion (31).

### Potential Application of NIRS in Sleep Researches

Over the last couple of decades, functional neuroimaging has been used to investigate neural mechanisms associated with the generation of sleep stages and pathophysiological mechanisms of sleep disordered (8, 32). The functional neural imaging, such as positron emission tomography (PET), transcranial Doppler (TCD), and functional magnetic resonance imaging (fMRI) have provided insight into cerebral metabolism, neuronal functioning, and vasomotor activity in terms of cerebral hemodynamics (3). These modalities provided global and regional brain activities during regular sleep cycles and sleep-disordered. However, they have limitations in terms of spatial and temporal resolution, safety and cost (33). A comparison of neuronal imaging techniques was shown in Table 1. PET has a limited temporal resolution, which fails to assess the transient changes of brain activities such as spindles or slow waves, and it has radiation stemming from the injection of radioactive isotopes. The high noise and restricted requirements of the fMRI environment limit the utilization of bedside monitoring, and the high cost is not suitable for routine monitoring. Regarding the indices detected by kinds of neuroimaging modalities, the cerebral hemodynamics underlying the regional tissues and blood components are different due to the different detection principles. fMRI is based on the principle of blood oxygen level-dependent (BOLD), which derives a change in local deoxy-hemoglobin resulting from the paramagnetic properties of deoxy-hemoglobin. It provides the mapping of neuroimaging of the entire brain. However, the change in HHb detected by fMRI can only reflect the change in the relative concentration of HHb before and after the event of interest. TCD provides a way to measure cerebral blood flow velocity in the basal brain arteries, which lacks the evaluation of the other parts of the brain. PET can measure the blood flow and glucose metabolism in the brain according to the injected radioactive tracer isotope. Moreover, the decay time of tracers was short, which could not realize the bedside sleep monitoring all night. NIRS is simple to set up and compatible with other techniques, such as EEG, and fMRI. It is not constrained by the environmental restrictions, such as fMRI or PET suffered from. NIRS has been validated against other neuroimaging modalities confirming that cerebral hemodynamics derived from NIRS correlated and were in sufficient agreement with the results derived from TCD, fMRI, PET, etc. (35–37). Although the deep tissue illumination depth is limited by NIRS (~2 cm) and is contaminated by the interferences of the extracerebral hemodynamics, these limitations do not affect the development and utilization of NIRS in academic studies and clinical scenarios. Considering the advantages of the NIRS including flexible application scenarios, suitable for specific populations (infants), and detection signals reflecting cerebral cortex cerebral oxygenation, cerebral blood volume, and cerebral perfusion, NIRS is important and has potential application in monitoring sleep activities at the bedside.

**Table 1:** Comparison between neuroimaging modalities (10, 32, 34).

**Technique**	**Origin**	**Strengths and limitations**
TCD	Blood circulation	Strengths: •Non-invasive; •Low cost; •Provide relative changes of cerebral blood flow velocity •Limitations: •Only measure basal arteries; •Low spatial resolution;
PET	Biochemistry activity	Strengths: •Provide whole brain scan at high spatial resolution; Limitations: •Invasive (request inject radioactive tracer); •Low temporal resolution;
EEG	Electrophysiological activity;	Strengths: •Non-invasive; •High temporal resolution; Limitations: •Low spatial resolution;
fMRI	Blood oxygenation	Strengths: •Non-invasive; •High spatial resolution; •Provide whole brain scan at high spatial resolution; •Highly reproducible and reliable; Limitations: •Discomfort of scanning environment; •Susceptible to motion artifacts; •Moderately expensive; •Merely reflect changes of de-oxyhemoglobin concentrations;
(f)NIRS	Blood oxygenation	Strengths: •Non-invasive; •Compatible with other modalities; •Safe and easy to set up; •Provide changes in oxy- and deoxy- hemoglobin concentrations; Limitations: •Merely cerebral cortex instead of deep structure; •Contaminated by the extracerebral hemodynamics;

The first area for the NIRS in the sleep filed is the application for sleep staging. Manuals of Rechtschaffen and Kales (R&K) were used as the guideline for visually scoring sleep since 1968. It divided sleep into seven distinct stages as follows: wake, stage 1, stage 2, stage 3, stage 4, stage REM, and movement time (38). Based on the R&K manual, the American Academy of Sleep Medicine (AASM) manual was proposed in 2007, as the standard guidelines for terminology, technical specifications, and scoring rules for sleep-related phenomena. According to the manual of AASM which was suitable for both adults and children, five sleep stages are defined (i.e., wakefulness (W), non-rapid eye movement (NREM) stage 1, stage 2, stage 3, and rapid eye movement (REM) (39). NREM stage 3 represents slow wave sleep (SWS) or deep sleep and is equivalent to the stage 3 and stage 4 of R&K. The scoring of each stage is based on the scoring rules for sleep-related phenomena, which correlate with changes in physiological signals, such as EEG, EOG, and EMG (40). One of the most apparent features of these phenomena is the variation in EEG waves. Previous functional neuroimaging studies have proved that the EEG rhythms underlying phasic events within specific sleep stages are associated with the activation or deactivation of corresponding cerebral regions (32). Because the increase and decrease of neural activity during sleep are accompanied by changes in cerebral oxygen demand and cerebral oxygen supply, this change can be reflected by the fluctuation of the NIRS signal. Continuous NIRS measurement contributes to a better understanding the interaction in cerebral blood volume (CBV), cerebral blood flow velocity (CBFV), and cerebral metabolic rate of oxygen (CMRO_2_) (41).

The second important research area of NIRS is related to sleep disorders. Sleep apnea syndromes have been associated with medical complications such as pulmonary and arterial hypertension, cardiovascular disease, excessive daytime sleeping, fatigue, morning headaches, and increased risk of cerebral infarction (42, 43). Three categories, obstructive, mixed, and central sleep apnea, are classified based on the criteria of respiratory events (44). Recognized as a major health concern, obstructive sleep apnea (OSA) syndrome is a chronic disease characterized by repeated partial or complete upper airway obstruction that leads to chronic intermittent hypoxia and sleep fragmentation during the night (45). OSA is associated with neurocognitive impairment, as the report that cognitive functions are altered in patients with OSA (46). OSA is also an independent risk factor for stroke (47). Hence, OSA-induced brain consequences have been of growing interest in the past few years (48). Moreover, continuous positive airway pressure (CPAP) therapy is used as a proper method to reduce the occurrence of OSA, however, it is necessary to determine its quantitative effectiveness. Therefore, it is important to understand the changes in the cerebral autoregulation mechanism under pathological conditions and the improvement in cerebral autoregulation during CPAP therapy.

## Hemodynamic Signal Variation in Human Sleep

**Figure 2:**
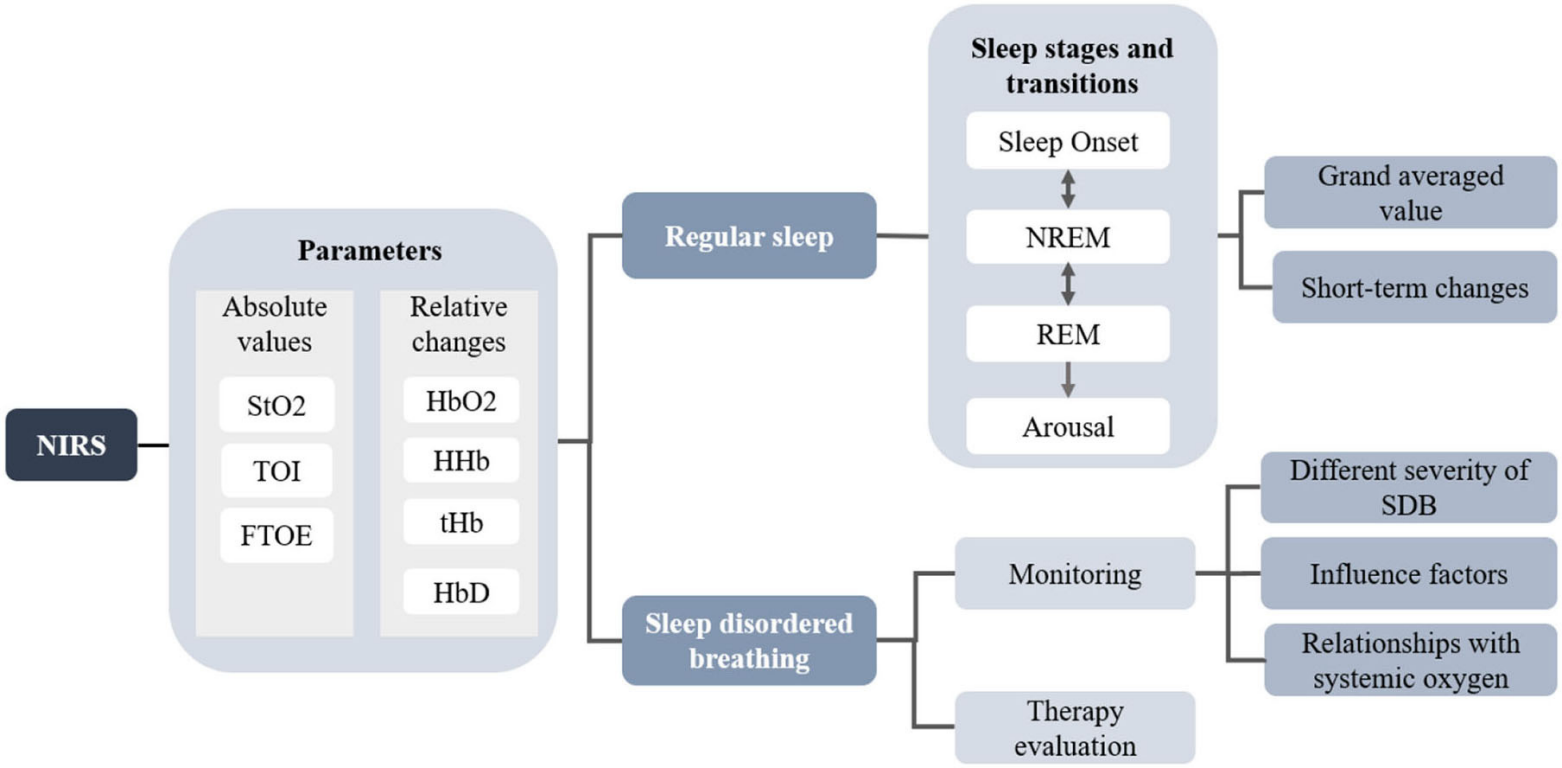
The diagram of the application of NIRS in human sleep studies.

Studies investigating the dynamic features of cerebrovascular perfusion and neurovascular coupling with synchronized detection of PSG and NIRS initiated in the 1990s. Hoshi and Livera first used NIRS to assess cerebral blood flow (CBF) and the cerebral oxygen metabolic rate (CMRO_2_) during nocturnal sleep and investigate the effect of hypoxemia and bradycardia on cerebral hemodynamics in preterm infants (49, 50). Regarding the investigation of cerebral hemodynamics during sleep, specific sleep events, that is, sleep stages and apneic events, annotated by the gold standard PSG or experienced clinicians, can serve as an instructor. Relevant cerebral hemodynamic features can then be extracted from the synchronous NIRS signals. The cerebral hemodynamic and oxygenation changes among different sleep events expressed as the changes of HbO_2_, HHb, tHb, HbD, and several derivatives (i.e., TOI, StO_2_, and FTOE), yield important brain activation information during sleep. The diagram of the application of NIRS is shown in Figure 2. A significant number of studies are dedicated to investigating the characteristic changes in cerebral hemoglobin concentration associated with sleep stages and sleep disorders. For regular sleep, researchers compared the cerebral hemodynamics among different sleep stages using grand averaged values of the specific sleep stages and investigated the transient hemodynamic patterns during stage transitions. The investigation of the short-term performance of NIRS signals may yield inconsistent results compared to the grand averaged method. Additionally, NIRS acted as an auxiliary tool to monitor the occurrence of sleep disordered breathing and as an auxiliary method to evaluate the treatment efficiency. According to the two main applications of NIRS in the sleep field, the studies investigating the fluctuation of cerebral hemodynamics are divided into the following two sections.

### Cerebral Hemodynamics in Regular Sleep Staging

**Figure 3:**
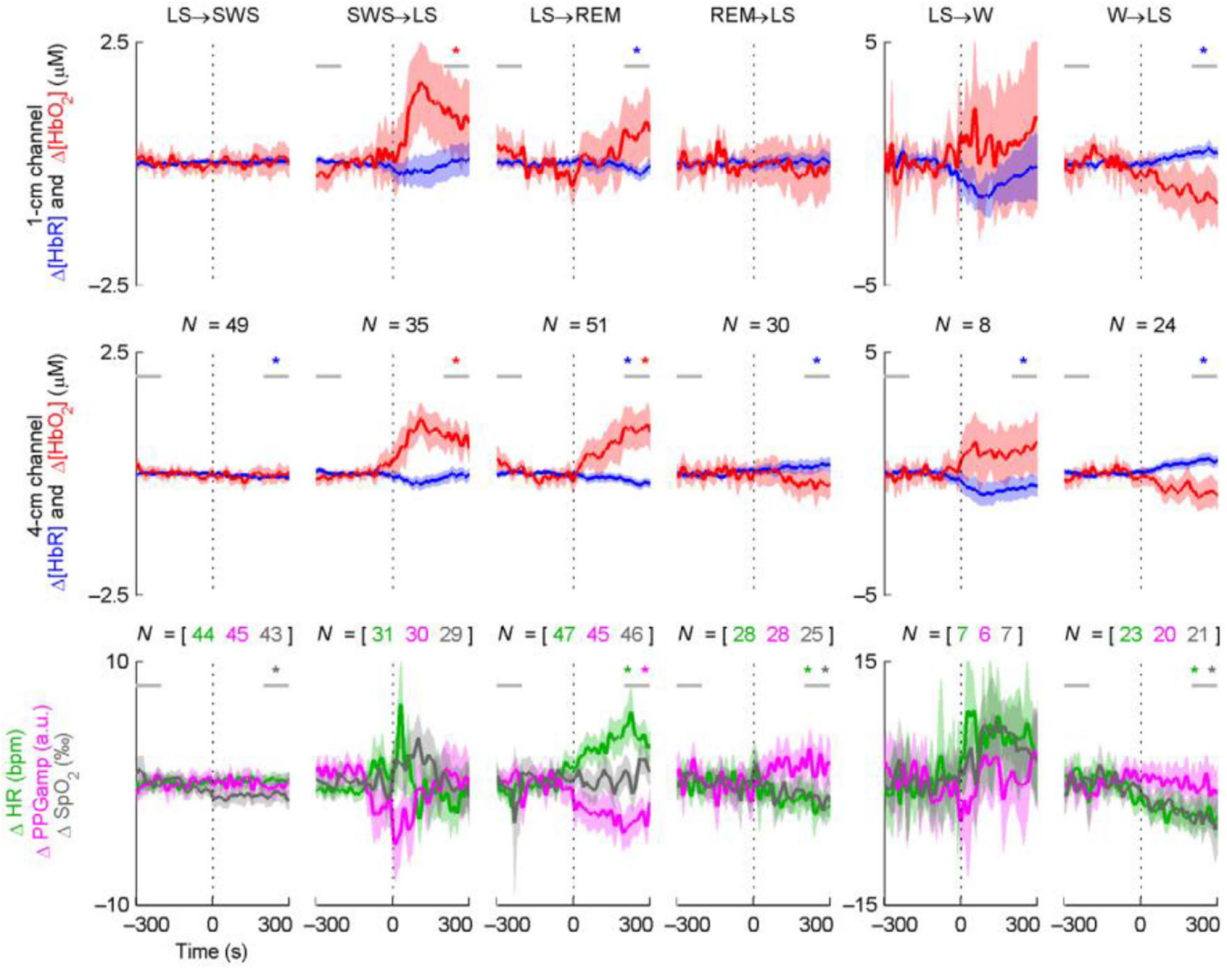
Time course averages around sleep stage transitions. *N* indicates the number of transitions (vertical dotted line) averaged and is equal for the two NIRS channels. The shading indicates the 95% confidence interval of the mean. Statistically significant baseline changes are indicated with a star of the corresponding color; the horizontal gray lines indicate the 100-s periods the baseline comparison was based on. The results from the baseline comparisons are not directly comparable to the confidence intervals, since paired *t*-tests were used for the former. For graphical purposes, LFOs were removed from the signals by low-pass filtering (passband edge at 40 mHz). This did not affect the statistical tests or the overall behavior of the curves. Note the three different transition types (persistent changes, transient changes, or no changes at all) and the asymmetry of opposing transitions in terms of the magnitude and speed of changes (e.g., LS→ WS vs. SW→ RLS). Δ[HbR] and PPGamp generally change in the opposite direction compared to Δ[HbO_2_] and HR. An example showing the changes of cerebral hemodynamics during the transition between different sleep stages adapted from Näsi et al. (34).

In earlier studies, the limited sampling rate of NIRS provided the opportunity to investigate the overall trend of sleep changes. The temporal trend of hemoglobin concentration changes provided us with basic information regarding hemodynamic changes during a sleep procedure. With the advancement of NIRS technology, the sampling rate has gradually increased, and multi-channel devices can simultaneously analyze changes in different brain regions. The increased sampling rate provided more detailed information about the phasic event within specific sleep stage [e.g., the transitions between light sleep (LS) and slow wave sleep (SWS) during NREM]. An example (34) showing the changes of cerebral hemodynamics during the transition between different sleep stages was show in Figure 3. During the signal pre-processing procedure, the awake state before sleep was used as a baseline in the NIRS study, and the changes in HbO_2_ and HHb detected by the CW-based system during sleep indicated the fluctuation of cerebral hemodynamics relative to the baseline. The specific changes in the NIRS signal in response to the distinct sleep stages were introduced as follows.

The sleep stage transition from wakefulness to sleep is usually accompanied by a subjective experience of self-awareness reduction. According to the temporal trend of NIRS signals, a decreasing trend in HbO_2_ and tHb, and an increasing trend in HHb was visually detected upon transitioning from wakefulness to sleep (50, 51). The results using grand averaged values of wakefulness and sleep were consistent with the visual inspection of the temporal trend during the initiation of sleep, showing larger values in HbO_2_ and tHb, and smaller values in HHb after entering sleep (34, 52–54). In the perspective of short-term changes in cerebral hemodynamics, NIRS has the capability to acquire transient changes in HbO_2_ and HHb during sleep stage transitions. Synchronized and parallel decreases in both HbO_2_ and HHb at a resolution of 5 s constructed the Switch Points after the points of Scored Sleep Onset (55).

With the progress of sleep stage, the sleep stages entered into NREM from wakefulness. The transitions between LS and SWS were exhibited during NREM. The grand averaged temporal values and power in the frequency domain presented suppression in SWS compared with other sleep stages (i.e., W, LS, and REM) (34, 56). An asymmetric change in terms of the short-term performance between LS and SWS exhibited a larger increase in HbO_2_, a decrease in HHb during the transitions from SWS to LS, and relatively constant state during the transitions from LS to SWS (34). In contrast, one study reported by Zhang using the grand averaged method presented increases in HbO_2_ and tHb during the SWS period compared to LS (54). Excluding the comparison of the NIRS parameters between LS and SWS during NREM sleep, significant details regarding neuronal activity were found during the NREM period. Cyclic alternating pattern (CAP) is a typical neuronal activity during the NREM state (57), which exhibits a fluctuation between phase A and phase B within one min (58). Phase A comprised the following three subtypes: A1, A2, and A3. The oscillations of hemodynamic signals corresponding roughly to the repetition rate of the CAP cycles were produced by alternating phases A and B, which exhibited a significant increase in HbO_2_, HHb, and tHb during phase A, especially in subtype A3, and thereafter decreased during the same cycle in B (58).

When entered into REM, transitions between REM and NREM recurrent appears. During the transition from NREM to REM, increases in HbO_2_ and tHb concomitant with a gradual decrease in HHb were observed in both studies evaluated by grand averaged values and transient changes (34, 50, 52, 53). Opposite and smaller changes with increased HHb and a slight decrease in HbO_2_ and tHb were observed during the transition from REM to NREM (34, 53). Significant differences in phase differences between HbO_2_ and HHb existed across sleep stages (24). The phase differences were found to progressively increase as the sleep stages processing fromwakefulness to the deeper sleep stages, and returned to the baseline when entered into REM.

Arousal from sleep is an important protective response to life-threatening events. A distinct change in NIRS parameters compared to sleep onset, namely an increase in HbO_2_ and tHb was observed for the sleep offset (55, 59). People also have the capability to wake up spontaneously at a desired time without receiving external stimuli. The increase in HbO_2_ accompanied a the decrease in delta power in the right prefrontal cortex ~30 min prior to self-wakening was shown, suggesting that the prefrontal cortex has the ability to estimate time (60).

TOI and StO_2_, the indicators for cerebral oxygenated saturation, were detected by spatially resolved spectroscopy (20). They provided absolute values instead of relative changes as HbO_2_ and HHb were detected by the CW-based NIRS system. Gradual decreases in TOI were exhibited as the sleep stages progressed from wakefulness to sleep, and from stage 1 to stage 2, as well as a reversed increase in TOI when entering REM from stage 2 (52). The temporal trend of StO_2_ presented a positive slope during the entire night using linear regression (59). In the short-time perspective of sleep transitions, the direction of changes in StO_2_ was the same as HbO_2_ which presented decreases upon sleep onset and during transitions from REM to NREM and an increase during transitions from NREM to REM (59). A comparison of the changes in StO_2_ between young adults and elderly subjects indicated that the StO_2_level was the same before sleep in both groups (53). However, it presented an opposite trend between the two groups after falling asleep. StO_2_ decreased upon the sleep onset in the elderly group but increased in young adults. When the subjects went into a deeper sleep, a further decrease in StO_2_ was observed in older subjects, whereas reversed changes were observed in the young adults.

Although studies regarding sleep in adolescents and adults have been conducted, no systematic comparison of the hemodynamic changes during sleep across different age groups has been made. Andreas et al. demonstrated that there was no significant difference in StO_2_ between adolescents and adults (53). Additionally, preterm and term infants are special populations, because of the immature autonomic regulation of cerebral blood flow and rapid development of cranial nerves in premature and term infants, the sleep structure and sleep events are different from those of adolescents and adults, such as the discontinuous EEG activities during quiet sleep (61). NIRS is particularly suitable for the study of infants because other methods cannot routinely be used without sedation (25). In preterm infants, an initial decrease in HbO_2_ with an opposite change in HHb and subsequent increased HbO_2_ were coupled with the burst of EEG activities during quiet sleep (62). Additionally, based on the studies as measured by PSG, failure to arouse may be one of the reasons for sudden infant death syndrome (63). The NIRS study in preterm infants found that tHb, HbD, and TOI, as well as heart rate and respiratory frequency remained constant during arousals (64). An investigation of the effects of sleep stage on cerebral hemodynamics in healthy term newborns found that cerebral FTOE, HbO_2_, and tHb increased significantly from active sleep to quiet sleep accompanied by a moderate decrease in HHb and reversed changes in tHb and HbO_2_ during the transitions from quiet to active sleep (65).

### Cerebral Hemodynamics in Sleep-Disordered Breathing

The investigation of cerebral hemodynamics in patients with sleep-disordered breathing syndromes provided significant clinical information regarding the effects of respiratory events on cerebral oxygenation. Typically, a pulse oximeter is used as the clinical method to evaluate sleep apnea in terms of the oxygenation by considering the changes in arterial oxygen saturation. Considering that changes in oxygenation may differ between tissues due to the difference in sensitivity to hypoxia, various studies have been conducted to assess the cerebral hemodynamic changes during nocturnal and daytime napping apneic episodes in age groups ranging from infancy to the elderly. Most studies have focused on infants and adults, and few have focused on children and the elderly. We summarized the studies into the four categories below.

#### Cerebral Hemodynamics Under Different Sleep-Disordered Breathing Events

The impact of cerebral hemodynamics on different sleep disordered breathing events has been investigated and compared in this review. The sleep-disordered breathing events include periodic breathing, apnea (central apnea, mixed apnea, and obstructive apnea), restless leg movement, differing severity of respiratory events, and breath hold test.

Due to the immature mechanism of autonomic regulation and the rapid development in the brain, newborns have a high risk of hypoxic cerebral injury and subsequently poor long-term neurodevelopmental outcomes, especially in ex-preterm infants (66). Period breathing is a unique and prevalent syndrome in pre-term and term infants compared to children or adults. Although the periodic breathing was benign and would disappear accompanying the maturation of the central nervous system with age (67), researchers concentrated on whether the periodic breathing would trigger fluctuations in cerebral hemodynamics and the patterns of cerebral oxygenation in response to periodic breathing. The results indicated that cyclical oscillations in cerebral hemodynamics were related to the occurrence of periodic breathing (67, 68). An increase in HHb and decreases in HbO_2_, HbD, StO_2_, or TOI were found in term and preterm infants (68–70); however, the changes in tHb were heterogeneous increasing in preterm infants and decreasing in term infants during periodic breathing (67, 68). Furthermore, Jenni et al. analyzed the causal relationship between periodic breathing and the fluctuation of CBV (67). They speculated that both periodic breathing and cyclical variations in CBV were mediated by the central nervous system, disproving the driving relationships between the two phenomena.

**Figure 4:**
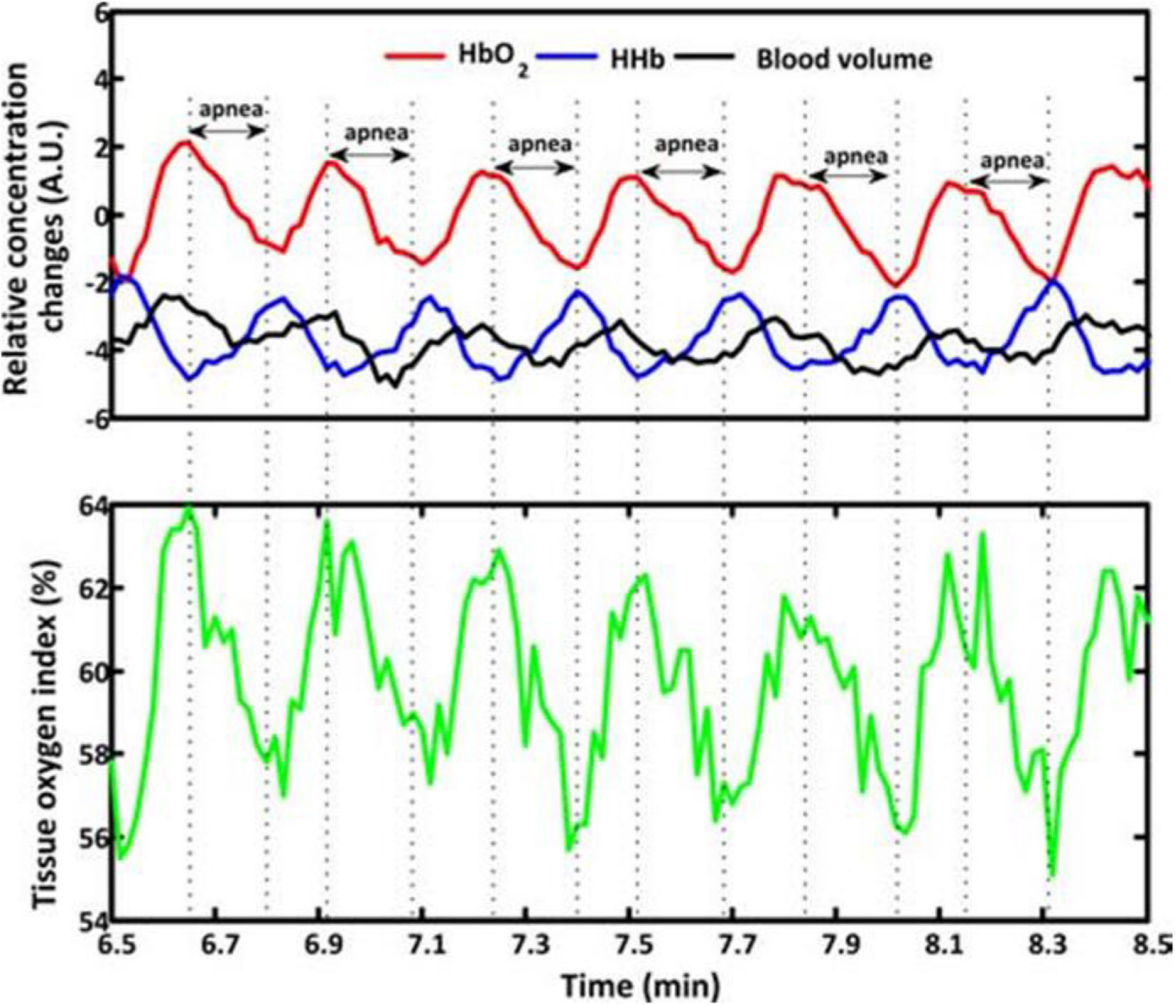
Typical fragments of cerebral hemodynamics during baseline measurements measured from Subject 1. The apnea events are marked by dash lines and double arrows. BV, HbO_2_ and HHb changes are expressed in arbitrary units (A.U.), as the mean value of first 60-s measurements was set at 0. During apnea events, the BV, HbO_2_ and tissue oxygen index signals show parallel decrease that are opposite to the HHb which increases after the initiation of apnea. Then an increase of BV, HbO_2_ and tissue oxygen index, while a decrease of HHb appears after the termination of apnea. An example of the changes of NIRS indices in response to apnea adapted from Zhang et al. (78).

Previous studies have investigated changes in NIRS parameters associated with different types of apnea. Significant oscillations of cerebral hemodynamics with declined HbO_2_, tHb, and TOI/StO_2_ accompanied by a synchronized increase in HHb were observed during the initiation of apneic episodes, and reverse changes of hemoglobin indices accompanied by inconsistent changes in tHb were obtained after the termination of apnea (71–79). An example of the changes of NIRS indices in response to apnea is shown in Figure 4. The fluctuation in NIRS was associated with the occurrence of apnea (77). However, NIRS in patients with OSA shows minor hemodynamic changes during apneic episodes and a subsequent significant decrease in tHb and HbO_2_ at the termination of the respiratory event (80). When comparing the effects of central, obstructive and mixed apnea on tHb in term and pre-term infants, all three types of apnea induced the following four distinct patterns of tHb: no change, isolated increase, isolated decrease, and both (81). It revealed that the OSA had the strongest impact on tHb compared to the other two types in the case of decreased tHb. Consistent results were found in adults and elderly subjects showing that subjects with OSA or severe SDB have more significant changes in NIRS (82, 83). Restless legs syndrome (RLS) with periodic limb movement during sleep is one of the common sleep disorders. An increased HbO_2_, tHb, and heart rate accompanied by heterogeneous changes in HHb were observed during RLS (79, 84). Owing to the distinct patterns in heart rate and CBV induced by OSA and RLS, the authors speculated that the occurrence of OSA and RLS were regulated by a different nervous system.

The change in NIRS is not only related to the type of apnea but also the severity and duration of apnea and desaturation degree of the pulse/arterial oxygen saturation. The severity of apnea was assessed using apnea-hypopnea index (AHI), which would affect the changes in NIRS (82). The threshold of AHI causing significant changes in cerebral hemodynamics was 30/h (82). A negative correlation was observed between StO_2_ and AHI (85), and the changes in the NIRS parameters significantly correlated with the duration of apnea in adults with OSA (75). Studies of different populations have found that various degrees of respiratory disorders have no apparent effect on TOI, FTOE and tHb (74, 86). However, a short duration of apnea accompanied by significantly reduced SpO_2_ results in significant changes in NIRS parameters (87), which validated that the severity of apnea cannot be evaluated by the isolated duration of apnea (88).

In previous studies, breath-holding in healthy subjects has been used to simulate the process of apnea and investigate the difference in the NIRS indices between breath-holding and apnea. Compared with the breath-holding group, the fluctuation magnitude of NIRS parameters during sleep was larger and the averaged absolute values in HbO_2_, tHb, and StO_2_ were significantly smaller in patients with OSA (77, 85). The canonical pattern with an increased HbO_2_ and decreased HHb was shown in healthy subjects during the breath-holding experiment (89). Although two distinct patterns of cerebral hemodynamics were observed in OSA patients, one presented a similar pattern to healthy subjects (80, 90) and the other was a reversed pattern when compared to healthy subjects during breath holding (89).

#### Physiological Factors Affecting Cerebral Hemodynamics During Sleep-Disordered Breathing

The consequence of changes in cerebral oxygenation during sleep-disordered breathing may be caused by complex physiological interactions. A few of studies discussed the factors which would affect cerebral oxygenation, including bradycardia, age, and sleep stages. Bradycardia, which is the main physiological parameter, is related to cardiac output and affects the oxygen delivery (91). Researchers have found that bradycardia with hypoxemia induces significant decreases in tHb and StO_2_ (49, 92). The changes in NIRS correlated with age. Premature and term infants with periodic breathing and apnea presented a decrease in TOI with increased age, and the reduction of TOI was greater in premature infants compared to full-term infants (69, 70, 93). Furthermore, the active sleep and spine position increases the incidence of periodic breathing and apnea in infants (69, 93). A positive correlation was found in StO_2_ with mean arterial pressure (MAP), arterial blood oxygen saturation (SaO_2_), age, and REM sleep stage in children with sleep-disordered breathing (71). Especially, NREM would augment the decline of TOI caused by SDB in children (73, 74), whereas the decline in NIRS was more pronounced during REM in adults (75, 76).

#### Comparison and Relations Between Cerebral Oxygenation and Systemic Oxygenation

As an indicator of peripheral capillary oxygen saturation detected by pulse oximeter and an indicator of cerebral oxygenation assessed by NIRS, the relationships between SpO_2_ and NIRS during apneic episodes attracted the attention of the researchers. Both techniques are sensitive to hypoxia induced by the pause of respiration, and the changes in SpO_2_ and cerebral oxygenation were found to be weakly correlated with small changes but strongly correlated in the case of for larger changes and severity SDB (77, 82, 94). The changes in SpO_2_ were several seconds later than NIRS (76, 77). A SpO_2_ threshold was found in preterm infants during apneic episodes, where SpO_2_ lowered to <85% would trigger a decrease in cerebral blood volume (95). Researchers have also quantified the changes in SpO_2_ and StO_2_, which reported that the desaturation of SpO_2_ was twice the reduction in StO_2_ (92, 94). There was no significant difference in CBV change for apnea with the same degree of desaturation at different durations of apnea. CBV decreased significantly when the apnea lasted for an extended period of time and was accompanied by a significant decrease in SpO_2_ (87). Notably, the values of StO_2_ remained above 60% for most infant subjects, despite having suffered severe SpO_2_ hypoxemia (92). The synchronized change in arterial oxygen saturation and cessation of airflow, as assessed by the apnea/hypopnea index (AHI) was the basic information for diagnosing apnea. The finding that AHI calculated by the StO_2_ was larger than that calculated by SpO_2_ (91), as well as the distinct patterns in the brain and muscles responding to intermittent hypoxia (96) verified that brain tissues were more sensitive to reduction in oxygen delivery induced by apnea.

#### The Evaluation of Treatment Efficacy of Positive Airway Pressure Therapy

Continuous positive airway pressure (CPAP) is the standard treatment for OSA. In addition to investigating the hemodynamic changes during sleep disorders, NIRS has the capability of evaluating the treatment effects of CPAP on obstructive sleep apnea. The occurrence of apneic episodes was accompanied by fluctuations in cerebral hemodynamics. The majority of studies have investigated the changes in cerebral tissue hemoglobin indices (HbO_2_, HHb, tHb) with and without CPAP or non-invasive ventilation (NIV) treatment in patients with OSA to evaluate the effect of treatment (72, 77, 78, 89, 97). Consistent reductions in the fluctuations of HbO_2_, HHb, and TOI were found in children and adults during CPAP. A sufficient treatment outcome and correcting cerebral oxygenation, further indicates that effective treatment may improve neurodevelopment. There was a significant improvement in behavioral problems and academic attention in patients with NIV treatment (72). Although efficient CPAP would suppress the fluctuation of NIRS and the occurrence of SDB, CPAP withdrawal after 2 weeks of therapy would result in the recurrence of OSA (97).

## Discussion of the Physiological and Pathological Mechanisms in Sleep Associated With NIRS

**Figure 5:**
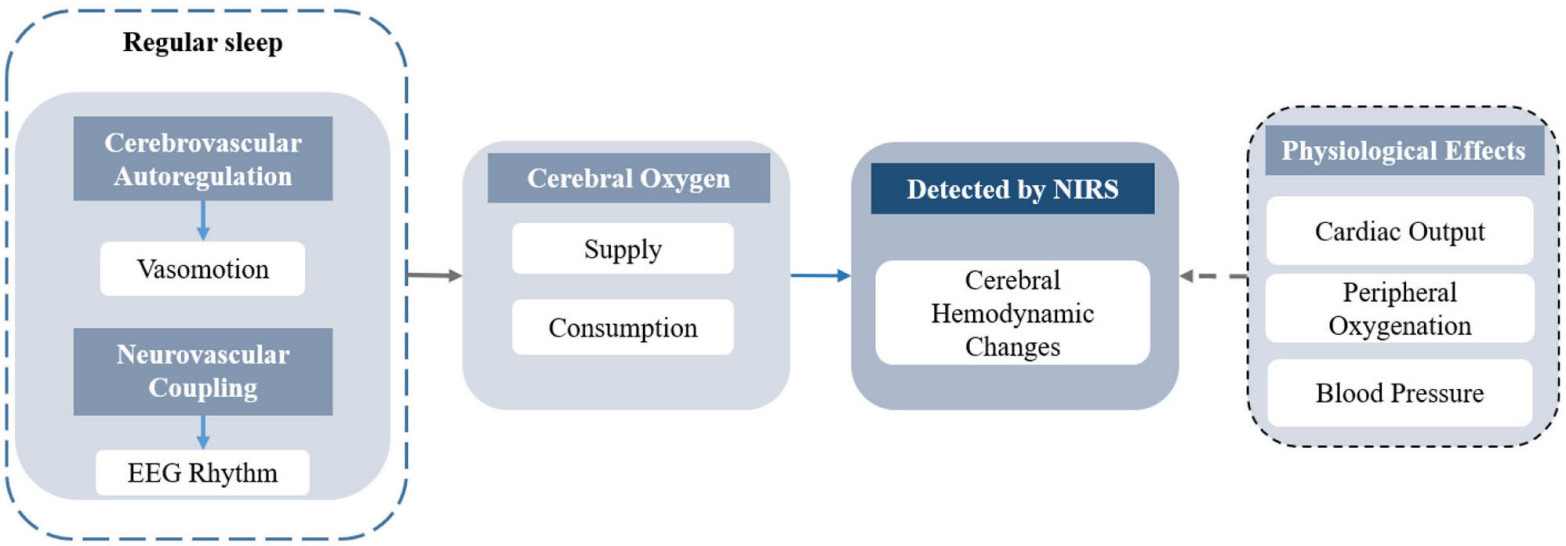
The diagram of the cerebral hemodynamics regulation in regular sleep.

The fluctuation of hemoglobin concentrations, induced by changes in sleep states or sleep disorders, reflects the different functional cerebral activities related to neuronal activities and physiological changes. A diagram of the cerebral hemodynamic regulations during regular sleep is presented in Figure 5. During regular sleep, the fluctuations in NIRS were coupled with the EEG rhythm and influenced by the interaction of the complex cerebrovascular regulatory mechanisms (98, 99). Although fluctuations in heart rate, blood pressure, and pulse oxygen saturation were reported during the sleep transitions (100, 101), the effect of these physiological parameters on the changes in cerebral hemodynamics needs further investigation (99). Based on neurovascular coupling, the changes in cerebral hemodynamics detected by NIRS reflect the interaction between cerebral oxygen consumption and cerebral oxygen supply. For instance, the occurrence of REM and arousal accompanied by the generation of dreams and the awakening of consciousness, respectively, indicate enhancement in neural activity, corresponding to the increases in HbO_2_ and tHb. With the reduction of self-awareness, the consistent decrease in HbO_2_, tHb, and TOI/StO_2_ during sleep onset reflect the reduction of CBF and CBV.

**Figure 6:**
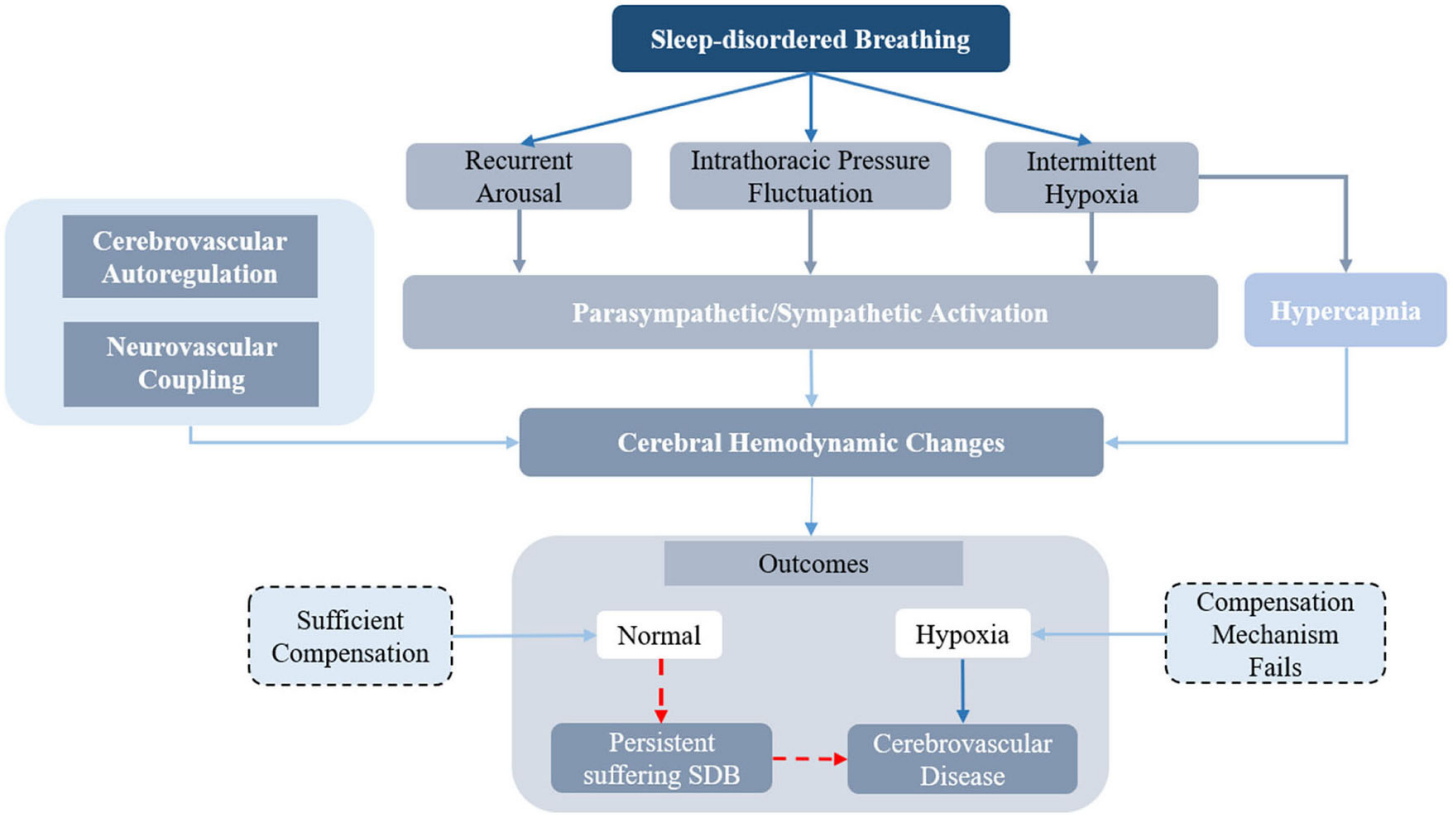
The diagram of the cerebral hemodynamics regulation in sleep disordered breathing.

In pathological conditions involving sleep-disordered breathing, the physiological changes induced by respiratory events will affect the cerebral hemodynamic changes and increase the risk of cerebrovascular disease. The flowchart of the cerebral hemodynamic regulation system in sleep disordered breathing is shown as Figure 6. The recurrent arousal, fluctuation of intrathoracic pressure, and intermittent hypoxia induced by the occurrence of apneic episodes would further influence parasympathetic/sympathetic activation. The complex physiological and pathological mechanisms, such as neuro-vascular coupling (80), sympathetic activity (90), and cerebral autoregulation (82, 92, 96) are responsible for the hemodynamic changes during the occurrence of sleep-disordered breathing. A sufficient compensating oxygen supply to the brain could protect the brain from hypoxia caused by apnea or periodic breathing. However, the persistence of SDB or severe apnea would disturb the regulation of cerebral hemodynamics and eventually lead to cerebrovascular disease. The cerebrovascular autonomic regulation mechanism and the regulation mechanism between peripheral oxygenation and cerebral oxygenation are presented below.

### Discussion of Cerebrovascular Autonomic Regulation Mechanism

The cerebrovascular autonomic regulation mechanism includes a wide range of cerebral blood flow regulation mechanisms, such as the neurovascular coupling. Neurovascular coupling is fundamental in NIRS studies; it is considered as the increase in cerebral blood flow induced by localized brain activity, and the increased blood supply is larger than the concomitant increase in oxygen consumption, resulting in a local increase in hemoglobin oxygenation (102). Therefore, the typical pattern of the NIRS signal responding to the neuronal activity presents an increased HbO_2_ with a decreased HHb, as shown in Figure 1, and the hemodynamic changes lag behind the neuronal activities (103). The neurovascular coupling mechanism provides sufficient explanation for the changes in blood flow and oxygenation stemming from neuronal activity during sleep periods. The increased HbO_2_ (i.e., REM and sleep offset) may be due to an increase in oxygen supply, determined by the increased cerebral blood flow, bringing hyper-oxygenated hemoglobin into the brain (62). In contrast, the decreased HbO_2_ (i.e., sleep onset and NREM) may be the consequence caused by the reduction of cerebral blood supply or maybe the reason for the decline in cerebral metabolism (54, 55). However, an inconsistent study indicated that the decrease in HbO_2_ during sleep onset reflected an unbalanced increase in cerebral metabolism and cerebral blood flow rather than a decline in cerebral metabolism (50). Both oxygen consumption and oxygen supply have an influence on the HHb concentration (104, 105), resulting in inconsistent variations with low amplitude. On the other hand, the time lags of cerebral hemoglobin concentrations behind the EEG rhythm fluctuations (52, 98) were consistent with the causal relations underlying neurovascular coupling. However, the EEG-NIRS time lag differs between sleep studies. Several studies have determined that hemodynamic changes are present prior to visible EEG activity (53, 54, 62). This may be due to the fact that PSG measurements with limited spatial information of EEG reflect global low-frequency synchronization of neural activity (106), and that NIRS has the capability of obtaining the local hemodynamics (11, 107), thus, the local hemodynamics occurring before global neural activity may be a hemodynamic marker of the measured cortex (54). Spontaneous oscillations of HbO_2_ and HHb are associated with regulated vasomotion (25, 26, 56, 108), and the fluctuations of HbO_2_ and HHb induced by SDB are partially related to cerebral vasomotor activity. Under normal circumstances, cerebral vessels regulate regional blood flow to accommodate changes in cerebral perfusion and arterial oxygenation, thereby maintaining the cerebral oxygen reserve at a fairly stable level (109). Vasomotion was identified by rhythmic oscillation in the tone of the blood vessels. Hemodynamic oscillations could reflect the vasomotion indirectly by the associated hemodynamic parameters such as blood flow, HbO_2_, and HHb (31, 110, 111). Cerebral vasomotion-induced hemodynamic oscillations are related to sleep stages, reflecting the variations in endothelial, neurogenic, and myogenic components of vasomotion detected by NIRS (56). In pathological conditions, the sustained elevation of cerebral blood flow is unique to the brain, thereby compensating for hypoxia and protecting the brain from anoxic injury (112). The cerebral vasodilatation induced by an increase in arterial carbon dioxide and subsequent decrease in cerebral resistance vessels may raise the cerebral blood volume, which reflects the increase in tHb (77, 110). The increased cerebral blood flow induced by hypopnea efficiently prevents the brain from severe hypoxia, and the pattern of cerebral hemodynamic changes is analogous to the canonical hemodynamic response to functional brain activities; namely, an increased HbO_2_ and tHb accompanied by a decreased HHb. However, the increased blood flow may not always be sufficient to compensate for oxygen desaturation, especially in patients with severe obstructive apneas (75, 77, 82). In patients with severe OSA, the failure of cerebral compensatory mechanisms cannot prevent the brain from hypoxia. The loss of cerebrovascular autoregulation is likely responsible for the inability of the brain to increase HbO_2_ in response to apnea events in patients with moderate to severe OSA. Additionally, similar patterns of cognitive performance between patients with OSA and patients with multi-infarct dementia verified that OSA is responsible for neuropsychological dysfunction (113). Therefore, the fluctuation of cerebral hemodynamics caused by apnea could affect normal sleep and disrupt cerebral autoregulation functions during sleep (78).

Sympathetic activity plays an important role in regulating cerebral hemodynamics in pathological conditions. Intrathoracic pressure swings and recurrent arousals induced by OSA would lead to increased sympathetic activation, and subsequently affect cortical cerebral vessels (114). The regulation between the cerebral blood volume and the whole-body blood volume was mediated by the sympathetic/parasympathetic system. Considering the composite of all information from the changes in cortical vascular LFO amplitude in animal and human models (115, 116), the increased cortical LFO amplitude was associated with increased sympathetic activation induced by hypoxia. However, there was no significant difference in the LFO amplitude between OSA patients and healthy controls during the resting state, suggesting that the influence of sympathetic activity induced by obstructive apneic episodes may be more pronounced during nocturnal sleep than during wakefulness. Contrary to the increase in tHb after the initiation of apnea events, several studies have indicated a decline in tHb during apneic episodes, especially in severe OSA (78). The decrement would be terminated by arousal, which was mediated by sympathetic activity and, subsequently, restored the airflow, resulting in an increase in cerebral CBV and HbO_2_ and a concomitant decrease in HHb (90).

### Discussion of the Regulation Mechanism Between Peripheral Oxygenation and Cerebral Oxygenation

The changes in peripheral oxygenation induced by sleep events reflect changes in arterial blood oxygen saturation (SaO_2_) and SpO_2_. The relationship between peripheral oxygenation and cerebral oxygenation was well-represented during regular and disordered sleep the regulation was associated with diverse factors, such as different populations. Accordingly, considering all these factors contributes to an all-around discussion on the topic of regulation.

Normal cerebral circulation relies on an intact cerebral autoregulation system to maintain adequate cerebral blood flow. In general, the values of SaO_2_ changed with sleep stages, specifically, it presented a decline when falling asleep (117–119). Once the SaO_2_ values fell below the normal level, regional vasodilatation would be evoked to compensate for desaturation. However, if cerebral blood flow increases, increased cerebral blood flow showing increased cerebral oxygenation can compensate for desaturation in SaO_2_ (73). The interaction between peripheral oxygenation and cerebral oxygenation is more prominent in pathological conditions. A majority of studies have found that the amount of decreased TOI is less than the reduction in SpO_2_ during apnea or hypopnea (72, 96, 97), which could be sufficiently explained by the complex mechanisms for modulating cerebral circulation. On the other hand, changes in the NIRS signal are a mixture of arterial (~10%), capillary (~20%), and venous (70%) contributions. The changes, predominantly depending on the veins, may result in a different response to the changes in oxygen supply/consumption compared to pulse oximetry. In addition to the incommensurate fluctuations in magnitude between SpO_2_ and cerebral hemodynamics, there is also a significant correlation between the changes in SpO_2_ and hemodynamics. A significant correlation between the changes in peripheral SpO_2_ and cerebral hemoglobin indices during sleep in OSA patients indicated that cerebral oxygenation was dependent on systemic oxygen delivery.

Moreover, the balance between peripheral and cerebral oxygenation was disrupted in several populations, for example preterm infants and the elderly (120, 121). The lower baseline SaO_2_ levels in the elderly would more likely suffer from mild-to-moderate hypoxemia and further influence the levels of cerebral oxygenation. It can also lead to more serious conditions such as cognitive impairment in the elderly (122). In preterm infants, the degree of cerebral oxygen desaturation was associated with a reduction in SpO_2_ induced by apneic episodes. The decline in cerebral oxygenation would affect the immature brain, which may cause irreversible brain damage. Apart from considering the significant reduction in SpO_2_ during apnea or hypopnea, researchers have suggested that a larger decline in TOI in response to central and obstructive events and periodic breath holding in children and infants compared to SpO_2_ indicated a better sensitivity of NIRS in evaluating cerebral hypoxia compared to peripheral oxygenation (74). Therefore, the use of NIRS as an adjunct to PSG would improve the ability to diagnose sleep apnea and better detect the effects of sleep apnea (72, 96, 97).

## Discussion of the Experimental Design and Signal Processing

The studies in section Hemodynamic Signal Variation in Human Sleep summarized the cerebral hemodynamic changes during sleep under different sleep events. There have been inconsistent results that would obfuscate the real cerebral oxygen supply in the course of sleep, resulting from the differences in experimental design, individual variation, and signal processing methods. The techniques that were used in the investigation of cerebral hemodynamics during sleep and the potential reasons for the discrepancy of the results are presented below.

### Discussion of Experimental Design

Commercial NIRS devices are mainly based on the following three techniques: continuous wave (CW), time domain (TD), and frequency domain (FD) system. Among the three techniques, the FD and TD methods require assessment of the path-length, thus obtaining the absolute changes in hemoglobin concentrations (123). The CW-based method cannot fully determine the optical properties of tissue, and therefore only relative changes of hemoglobin concentrations can be detected (11). Furthermore, the spatially resolved spectroscopy could obtain the absolute values, i.e., TOI and StO_2_. The structure of instruments differs between studies, resulting in cerebral hemodynamic activation variance.

Most sleep studies used NIRS to detect cerebral hemodynamic changes over the forehead. Neuroimaging and electrophysiological studies have indicated that the mediation of sleep and dreaming requires the participation of various cerebral regions, including the brainstem, thalamus, basal forebrain, and so on (124). The prefrontal cortex is implicated in executive control, personal expression, and planning complex cognitive behavior (125). In particular, the function of the prefrontal cortex is sensitive during sleep, which indicates early changes in the transition from wakefulness to sleep and exhibits a deactivation during NREM compared with waking (32, 126, 127). Additionally, the prefrontal cortex contributes to controlling the timer rate, attention allocation, and temporal memory processing (128). Therefore, the investigation of the prefrontal hemodynamics may provide more information regarding slow specific temporal activation patterns during sleep. Furthermore, the hair on the scalp significantly attenuates light and reduces the signal-to-noise ratio. In neonates, the temporal areas are rather flat and most sensitive to hypoxia during growth. Premature infants, particularly, are susceptible to brain impairment on the left side of the temporal areas. Although it is convenient for the long-term monitoring of cerebral hemodynamic changes in the prefrontal region during sleep, the different patterns and activation in different cerebral regions (51, 52, 98) indicate that it is necessary to monitor different cerebral regions using multiple channels.

Studies investigating cerebral hemodynamics during normal sleep were conducted in both nocturnal sleep and daytime napping. To shorten sleep latency, subjects were restricted to sleep deprivation for several hours from their regular sleep time, especially in daytime experiments (55). Subjects experienced 1–2 sleep cycles due to the limited duration of experiment. Additionally, the guidelines for sleep stage scoring are different, and the rules of R&K and the manual of AASM were commonly used. Comparing the two guidelines for sleep stage scoring, the major differences involve the definition of sleep–wake transition, sleep state terms, and the recording method (40). The different sleep structures for the sleep stages may lead to discrepant results. Furthermore, the syndrome or severity of sleep disorders including snoring, periodic breathing, sleep apnea (obstructive sleep apnea, central apnea, and mixed sleep apnea), and the inclusion criteria of subjects (i.e., AHI value and the reduction of SpO_2_), differ between studies. Heterogeneous patients with sleep disordered accompanied by abnormal cardiac function or blood pressure may further influence cerebral hemodynamics (71, 96).

NIRS, as a potential bedside monitoring tool, shows an advantage in assessing the hemodynamic changes of the cerebral cortex. However, one major disadvantage of NIRS is that the changes in cerebral hemodynamics are significantly affected by the extracerebral tissue contaminants (such as the scalp). The prevalent method used to reduce scalp hemodynamics is performed using a multi-detector at varying distances from the source. The short distance primarily scalp hemodynamics, and the other distance measures both the scalp and cerebral hemodynamics. NIRS signals obtained from a short distance subtracted from the larger one receive a corrected NIRS (129). Although the predominant changes in the NIRS signal are the result of changes in cerebral hemodynamics, the extracerebral hemodynamic changes affect the outcome to some extent (130).

### Discussion of Signal Processing Method

**Figure 7:**
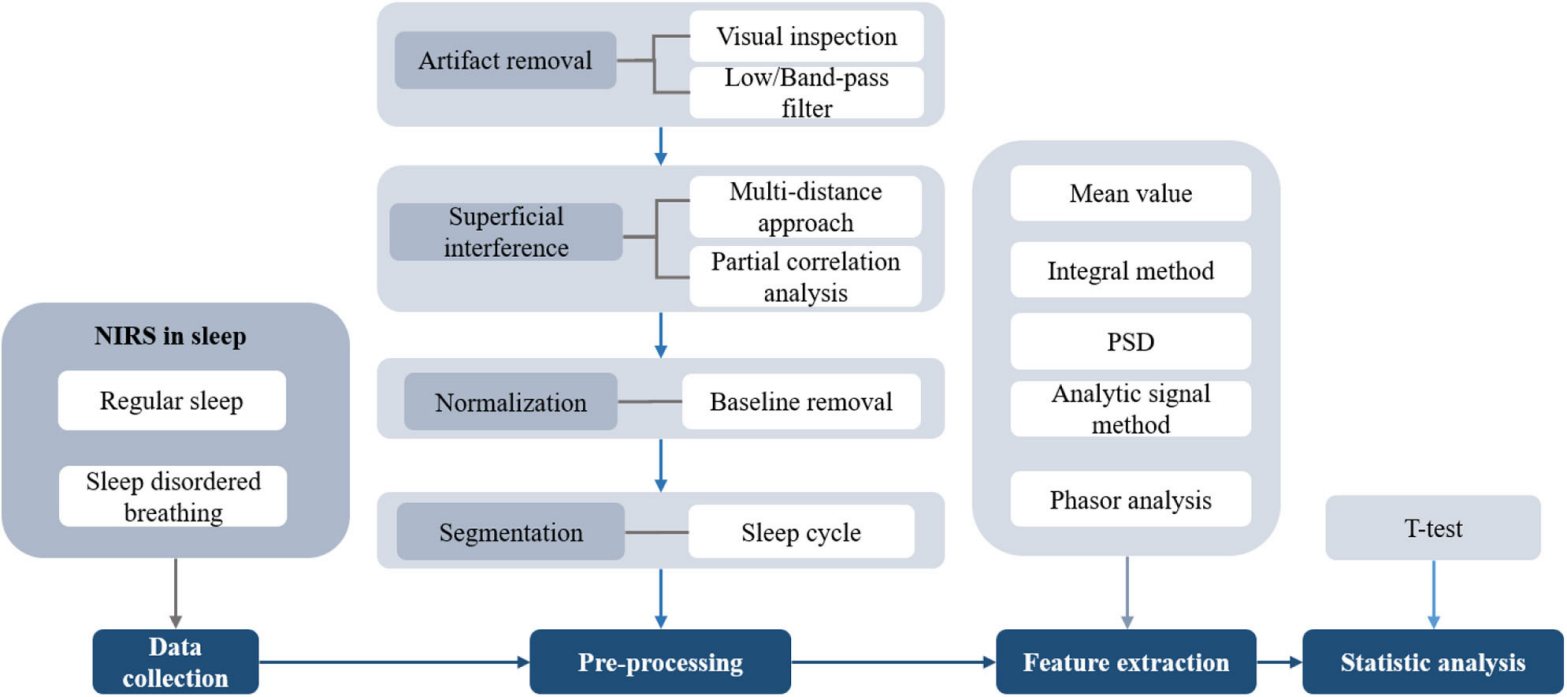
The flowchart of signal processing in sleep using NIRS.

The standard procedure for signal processing during sleep is shown in Figure 7. Motion artifacts are often a significant component of the measured NIRS signals. The magnitude of the motion artifacts is typically significantly larger than that of the NIRS signals. To properly interpret the changes in cerebral hemodynamics, motion artifacts should be detected and removed (131). In general, the following two distinct methods are used to eliminate or reduce motion artifacts: a method with an additional input (e.g., inertial measurement unit) and a method that does not require additional sensors (e.g., Wiener filter and wavelet-based filtering). Details regarding the motion artifact correction techniques can be found in previous studies (132). In sleep studies using NIRS, motion artifacts are usually removed directly with visual inspection, and subsequently, low-pass or band-pass filters are used to extract the signals of interest.

One of the disadvantages of NIRS is that, the detected NIRS signals mixed with the scalp blood flow signals could interfere with cortical hemodynamic signals of interest. A common method eliminates the interference of superficial tissue by using the multi-distance approach (56). Different patterns in HHb were found between the two distances of NIRS signals (58). Moreover, the non-correlation between NIRS changes and MAP changes represent the intact cerebrovascular autoregulation (133). One proposed method is partial correlation analysis (98). The relationships between EEG and NIRS could be better reflected after eliminating the interference of MAP on cerebral hemodynamics. Most studies have focused on the changes in HbO_2_ mainly due to the apparent variation in response to brain activities. Additionally, the investigation of the changes in tHb, StO_2_, and TOI was prevalent in clinical situations, as the robust property for motion artifacts and visualized the absolute values (23). The parameters commonly used and the proportion of the NIRS parameters in the sleep studies are summarized in Table 2.

**Table 2:** List of the most reported parameters in sleep studies using NIRS (12) and the proportion of their utilization.

**Abb**.	**Parameter**	**Interpretation for physiology**	**Proportion**
HbO_2_	Oxygen hemoglobin concentration	Changes in cerebral hemodynamics and blood oxygenation	++
HHb	De-oxygen hemoglobin concentration	Changes in cerebral hemodynamics and blood oxygenation	++
tHb	Total hemoglobin concentration	Marker for cerebral blood volume	++
HbD	Hemoglobin oxygenation index (HbO_2_-HHb)	Changes of cerebral oxygenation	–
TOI	Cerebral tissue oxygenation index	Absolute value to assess oxygenation	+
StO_2_	Regional cerebral oxyhemoglobin saturation	Absolute value to assess oxygenation	+
FTOE	Fractional tissue oxygen extraction	Ratio of cerebral oxygen consumption to delivery	–

*++: prevalent used. +: commonly used. –: less used*.

In data preprocessing, the standard of data segmentation is different. Sleep time included sleep procedures during the entire night, daytime napping, or only included the first sleep cycle (52, 54). In the time series analysis techniques, the NIRS signal was first normalized to obtain the relative changes according to the principle of CW-based NIRS. Then, the baseline corrected data were divided into multiple segments of interest with the time windows. The window length ranged from several seconds to several minutes. The data were analyzed by averaging segmentations of NIRS or the transient activation time-locked to the shift of EEG burst. To compare the significant changes in the difference of cerebral hemodynamics in response to sleep events, *t*-tests and analysis of variance were used. Thus, the selection criteria of the time windows cause inconsistent results, such as the synchronized and reciprocal changes of HbO_2_ and HHb at sleep onset using short-term analysis and the mean values of sleep stages, respectively (54, 55). The insufficient signal-to-noise ratio (SNR) stemming from the limited sleep segmentations or subjects may not be able to extract a representative pattern for specific sleep events. Furthermore, the sleep stages are labeled based on the PSG results, which require an analysis using an epoch of 30 s. The inconsistent result may be due to the limited time resolution caused by the annotation (55).

There was no standardized method for extracting the cerebral hemodynamic parameters. The integral method which calculates the integrals of changing curve in NIRS, proposed by Pizza (82), is a useful tool for analyzing the NIRS signals, which considers the duration of the event and suppresses any artifact interferences (79). In addition to the temporal trends in cerebral hemodynamics, studies using the power spectral density (PSD), analytic signal method and phasor analysis provide a better way to interpret the intrinsic hemodynamic oscillations during sleep (24, 56). The phasor representation, which decomposed the changes in hemoglobin concentrations into CBV and CBFV, provided a new way to investigate the physiological origin of the oscillations in HbO_2_ and HHb (24).

## Future Perspectives

The benefit of combining EEG with fNIRS during sleep in neuroscientific research and clinical applications provided a comprehensive understanding of the interaction between the nervous system and cerebral vasculature. The investigation of spontaneous cerebral hemodynamic oscillations with NIRS provides novel information regarding the neurophysiological features of sleep. In this paper, we reviewed previous sleep studies that utilized NIRS in both regular sleep cycles and pathological conditions with sleep disorders. However, the relationship between oxygen supply, oxygen consumption, and oxygen perfusion in a specific sleep state is still unknown. Regarding the complex interactions between CBF, CBV, CBFV, and cerebral perfusion pressure, conflicting conclusions on the causality between oxygen metabolic rate, oxygen consumption, neuronal activity, and hemodynamic changes should be further investigated.

During regular sleep cycles, the fluctuation of cerebral hemodynamics was correlated with the transitions of sleep stages (fluctuation in specific EEG-wave). NIRS utilization in sleep studies provides insight into functional neuroanatomy by comparing the changes between different sleep stages and transient fluctuations during sleep transitions benefiting from the relatively higher temporal resolution compared with other neuroimaging techniques. The significant fluctuation of cerebral hemodynamics during the awakening period increased the risk of cerebrovascular disease due to the immature brain autoregulation ability in premature infants and the decline of autonomic regulation ability in the elderly. Therefore, bedside monitoring is particularly important for these illumination depth of populations. The disruption of normal nocturnal sleep induced by apnea leads to fluctuations of cerebral hemodynamics, which jeopardizes the cerebral autoregulation mechanisms and ultimately increases the risk of cerebrovascular disease. Fortunately, CPAP treatment can reduce or eliminate airway obstruction, thereby suppressing cerebral hemodynamic fluctuations. Therefore, NIRS has potential application prospects in the monitoring and treatment evaluation of sleep disorders. Additionally, studies investigating impaired cerebral blood flow and cerebrovascular reactivity associated with sleep disorders should be noted and used for developing a personalized effective CPAP treatment utilizing NIRS.

Although NIRS is a promising tool for investigating cerebral hemodynamics during sleep, there are also limitations that need to be further explored. The experimental design is essential for acquiring robust signals, but it differs among existing studies. Neuroscientific research and clinical trials were conducted during daytime napping or nocturnal sleep, and the population studied ranged from premature to elderly, and the sleep disorders ranged from mild to severe. In future experiments, the unification of the age of the populations and the severity of the disease, as well as the inclusion of an adequate number of subjects, should be considered.

Furthermore, the development of the brain during childhood is sequential (134) and the discrepancy results across cerebral regions, suggesting the significance of monitoring in multi-channels. The standardized method for signal processing is not well-established. Notably, the interference of the extracerebral hemodynamics on the NIRS signal needs to be considered when extracting cerebral hemodynamic responses. The traditional signal processing method for NIRS sleep is the study of the trend in NIRS signal changes. It is necessary to find effective methods for signal analysis and to extract efficient features. Additionally, the insufficient sampling rate of the existing instruments limits the potential role of low frequency oscillations of NIRS in investigating cerebral vasomotions, which play an essential role in regulating cerebral blood perfusion. Inspired by the multimodal neuroimaging utilized in studying and diagnosing pathologies (135) and the simultaneous EEG-NIRS measurements used in the context of BCI (136), the fusion of NIRS-EEG bimodal signals has potential applications in automatic sleep staging, automatic diagnosis of sleep disorders, and evaluation of therapeutic effects.

## Author Contributions

HR, XJ, and KX reviewed the literatures and drafted the manuscript. CC, YY, CD, and WC revised and proofread the manuscript. All authors read and approved the final manuscript.

## Conflict of Interest

The authors declare that the research was conducted in the absence of any commercial or financial relationships that could be construed as a potential conflict of interest.

## Abbreviations

NIRS: Near-infrared spectroscopy
PSG: polysomnography
EEG: electroencephalography
EMG: electromyography
EOG: electrooculogram
BCI: brain-computer interface
CW: continuous-wave
FD: frequency-domain
TD: time-domain
MBLL: modified Beer-Lambert law
fMRI: functional magnetic resonance imaging
PET: positron emission tomography
TCD: transcranial Doppler
HbO_2_: oxygen hemoglobin concentration
HHb: deoxy-hemoglobin concentration
tHb: total hemoglobin concentration
HbD: hemoglobin difference
StO_2_: brain tissue oxygen saturation
TOI: tissue oxygenation index
FTOE: fractional tissue oxygen extraction
SaO_2_: arterial blood oxygen saturation
SpO_2_: peripheral capillary oxygen saturation
LFO: low frequency oscillations
VLFO: very low frequency
R&K: Rechtschaffen and Kales
AASM: American Academy of Sleep Medicine
CBF: cerebral blood flow
CBV: cerebral blood volume
CBFV: cerebral blood flow velocity
CMRO_2_: cerebral metabolic rate of oxygen
MAP: mean arterial pressure
SDB: sleep-disordered breathing
OSA: obstructive sleep apnea
W: wakefulness
LS: light sleep
SWS: slow wave sleep
REM: rapid eye movement
NREM: non-rapid eye movement
CAP: cyclic alternating pattern
CPAP: continuous positive airway pressure
NIV: non-invasive ventilation
AHI: apnea-hypopnea index
PSD: power spectral density.

## References

[1] Otte A Turkheimer F Rosenzweig I All you need is sleep. EBioMedicine. (2016) 12:2–3. 10.1016/j.ebiom.2016.10.003 27729215 PMC5078613

[2] Heinzer R Vat S Marques-Vidal P Marti-Soler H Andries D Tobback N Prevalence of sleep-disordered breathing in the general population: the HypnoLaus study. Lancet Respir Med. (2015) 3:310–8. 10.1016/S2213-2600(15)00043-0 25682233 PMC4404207

[3] Raven F Van der Zee EA Meerlo P Havekes R The role of sleep in regulating structural plasticity and synaptic strength: implications for memory and cognitive function. Sleep Med Rev. (2018) 39:3–11. 10.1016/j.smrv.2017.05.002 28641933

[4] Beaudin AE Waltz X Hanly PJ Poulin MJ. Impact of obstructive sleep apnoea and intermittent hypoxia on cardiovascular and cerebrovascular regulation. Exp Physiol. (2017) 102:743–63. 10.1113/EP086051 28439921

[5] Fox MD Raichle ME. Spontaneous fluctuations in brain activity observed with functional magnetic resonance imaging. Nat Rev Neurosci. (2007) 8:700–11. 10.1038/nrn2201 17704812

[6] Zoccoli G Walker AM Lenzi P Franzini C. The cerebral circulation during sleep: regulation mechanisms and functional implications. Sleep Med Rev. (2002) 6:443–55. 10.1053/smrv.2001.0194 12505477

[7] Ayalon L Peterson S. Functional central nervous system imaging in the investigation of obstructive sleep apnea. Curr Opin Pulmonary Medi. (2007) 13:479. 10.1097/MCP.0b013e3282f0e9fb 17901752

[8] Desseilles M Dang-Vu T Schabus M Sterpenich V Maquet P Schwartz S. Neuroimaging insights into the pathophysiology of sleep disorders. Sleep. (2008) 31:777–94. 10.1093/sleep/31.6.777 18548822 PMC2442420

[9] Hossmann KA. The hypoxic brain. Insights from ischemia research. Adv Exp Med Biol. (1999) 474:155–69. 10.1007/978-1-4615-4711-2_14 10635000

[10] Ferrari M Quaresima V. A brief review on the history of human functional near-infrared spectroscopy (fNIRS) development and fields of application. Neuroimage. (2012) 63:921–35. 10.1016/j.neuroimage.2012.03.049 22510258

[11] Scholkmann F Kleiser S Metz AJ Zimmermann R Mata Pavia J Wolf U. A review on continuous wave functional near-infrared spectroscopy and imaging instrumentation and methodology. Neuroimage. (2014) 85(Pt 1):6–27. 10.1016/j.neuroimage.2013.05.004 23684868

[12] Obrig H. NIRS in clinical neurology - a “promising” tool? Neuroimage. (2014) 85(Pt 1):535–46. 10.1016/j.neuroimage.2013.03.045 23558099

[13] Jöbsis FF. Noninvasive, infrared monitoring of cerebral and myocardial oxygen sufficiency and circulatory parameters. Science. (1977) 198:1264–7. 10.1126/science.929199 929199

[14] Obrig H Villringer A. Beyond the visible–imaging the human brain with light. J Cereb Blood Flow Metab. (2003) 23:1–18. 10.1097/01.WCB.0000043472.45775.29 12500086

[15] Delpy DT Cope M Quantification in tissue near-infrared spectroscopy. Philos Trans R Soc Lond B Biol Sci. (1997) 352:649–59. 10.1098/rstb.1997.0046

[16] Quaresima V Ferrari M Functional near-infrared spectroscopy (fNIRS) for assessing cerebral cortex function during human behavior in natural/social situations: a concise review. Organ Res Methods. (2019) 22:46–68. 10.1177/1094428116658959

[17] De Blasi RA Almenräder N Ferrari M. Brain oxygenation monitoring during cardiopulmonary bypass by near infrared spectroscopy. Adv Exp Med Biol. (1997) 413:97–104. 10.1007/978-1-4899-0056-2_11 9238490

[18] Vernieri F Rosato N Pauri F Tibuzzi F Passarelli F Rossini PM. Near infrared spectroscopy and transcranial Doppler in monohemispheric stroke. Eur Neurol. (1999) 41:159–62. 10.1159/000008041 10202248

[19] Tsuji M duPlessis A Taylor G Crocker R Volpe JJ. Near infrared spectroscopy detects cerebral ischemia during hypotension in piglets. Pediatr Res. (1998) 44:591–5. 10.1203/00006450-199810000-00020 9773851

[20] Suzuki S Takasaki S Ozaki T Kobayashi Y Tissue oxygenation monitor using NIR spatially resolved spectroscopy. In: Optical tomography and spectroscopy of tissue III. Vol. 3597 International Society for Optics and Photonics (1999). p. 582–92.

[21] Quaresima V Sacco S Totaro R Ferrari M. Noninvasive measurement of cerebral hemoglobin oxygen saturation using two near infrared spectroscopy approaches. J Biomed Opt. (2000) 5:201–5. 10.1117/1.429987 10938784

[22] Yoshitani K Kawaguchi M Tatsumi K Kitaguchi K Furuya H. A comparison of the INVOS 4100 and the NIRO 300 near-infrared spectrophotometers. Anesth Analg. (2002) 94:586–90. 10.1097/00000539-200203000-00020 11867380

[23] van Bel F Lemmers P Naulaers G. Monitoring neonatal regional cerebral oxygen saturation in clinical practice: value and pitfalls. Neonatology. (2008) 94:237–44. 10.1159/000151642 18784420

[24] Pierro ML Sassaroli A Bergethon PR Ehrenberg BL Fantini S. Phase-amplitude investigation of spontaneous low-frequency oscillations of cerebral hemodynamics with near-infrared spectroscopy: a sleep study in human subjects. Neuroimage. (2012) 63:1571–84. 10.1016/j.neuroimage.2012.07.015 22820416 PMC3472105

[25] Taga G Konishi Y Maki A Tachibana T Fujiwara M Koizumi H. Spontaneous oscillation of oxy- and deoxy- hemoglobin changes with a phase difference throughout the occipital cortex of newborn infants observed using non-invasive optical topography. Neurosci Lett. (2000) 282:101–4. 10.1016/S0304-3940(00)00874-0 10713406

[26] Obrig H Neufang M Wenzel R Kohl M Steinbrink J Einhäupl K. Spontaneous low frequency oscillations of cerebral hemodynamics and metabolism in human adults. Neuroimage. (2000) 12:623–39. 10.1006/nimg.2000.0657 11112395

[27] Wolf M Wolf U Toronov V Michalos A Paunescu LA Choi JH. Different time evolution of oxyhemoglobin and deoxyhemoglobin concentration changes in the visual and motor cortices during functional stimulation: a near-infrared spectroscopy study. Neuroimage. (2002) 16:704–12. 10.1006/nimg.2002.1128 12169254

[28] Zheng F Sassaroli A Fantini S. Phasor representation of oxy- and deoxyhemoglobin concentrations: what is the meaning of out-of-phase oscillations as measured by near-infrared spectroscopy? J Biomed Opt. (2010) 15:040512. 10.1117/1.3483466 20799778 PMC2941517

[29] Novi SL Rodrigues RBML Mesquita RC. Resting state connectivity patterns with near-infrared spectroscopy data of the whole head. Biomed Opt Express. (2016) 7:2524–37. 10.1364/BOE.7.002524 27446687 PMC4948611

[30] Niu H He Y. Resting-state functional brain connectivity: lessons from functional near-infrared spectroscopy. Neuroscientist. (2014) 20:173–88. 10.1177/1073858413502707 24022325

[31] Cui R Zhang M Li Z Xin Q Lu L Zhou W. Wavelet coherence analysis of spontaneous oscillations in cerebral tissue oxyhemoglobin concentrations and arterial blood pressure in elderly subjects. Microvasc Res. (2014) 93:14–20. 10.1016/j.mvr.2014.02.008 24594440

[32] Dang-Vu TT Schabus M Desseilles M Sterpenich V Bonjean M Maquet P. Functional neuroimaging insights into the physiology of human sleep. Sleep. (2010) 33:1589–603. 10.1093/sleep/33.12.1589 21120121 PMC2982729

[33] Maquet P. Functional neuroimaging of normal human sleep by positron emission tomography. J Sleep Res. (2000) 9:207–31. 10.1046/j.1365-2869.2000.00214.x 11012860

[34] Näsi T Virtanen J Noponen T Toppila J Salmi T Ilmoniemi RJ. Spontaneous hemodynamic oscillations during human sleep and sleep stage transitions characterized with near-infrared spectroscopy. PLoS ONE. (2011) 6:e25415. 10.1371/journal.pone.0025415 22043284 PMC3197192

[35] Huppert TJ Hoge RD Diamond SG Franceschini MA Boas DA. A temporal comparison of BOLD, ASL, and NIRS hemodynamic responses to motor stimuli in adult humans. NeuroImage. (2006) 29:368–82. 10.1016/j.neuroimage.2005.08.065 16303317 PMC2692693

[36] Rivera-Lara L Geocadin R Zorrilla-Vaca A Healy R Radzik BR Palmisano C. Validation of near-infrared spectroscopy for monitoring cerebral autoregulation in comatose patients. Neurocritical Care. (2017) 27:362–9. 10.1007/s12028-017-0421-8 28664392 PMC5772737

[37] Rostrup E Law I Pott F Ide K Knudsen GM. Cerebral hemodynamics measured with simultaneous PET and near-infrared spectroscopy in humans. Brain Res. (2002) 954:183–93. 10.1016/S0006-8993(02)03246-8 12414101

[38] Hori T Sugita Y Koga E Shirakawa S Inoue K Uchida S. Proposed supplements and amendments to 'A manual of standardized terminology, techniques and scoring system for sleep stages of human subjects', the Rechtschaffen & Kales (1968) standard. Psychiatry Clin Neurosci. (2001) 55:305–10. 10.1046/j.1440-1819.2001.00810.x 11422885

[39] Malhotra RK Avidan AY Sleep stages and scoring technique. In: Atlas of Sleep Medicine. Elsevier. p. 77–99.

[40] Moser D Anderer P Gruber G Parapatics S Loretz E Boeck M. Sleep classification according to AASM and Rechtschaffen & Kales: effects on sleep scoring parameters. Sleep. (2009) 32:139–49. 10.1093/sleep/32.2.139 19238800 PMC2635577

[41] Fantini S Aggarwal P Chen K Franceschini MA Ehrenberg BL Nearinfrared spectroscopy and polysomnography during all-night sleep in human subjects. In: Tuchin V, editor. Saratov Fall Meeting 2002: Optical Technologies in Biophysics and Medicine IV. Saratov: International Society for Optics and Photonics p. 155–62.

[42] Guilleminault C Tilkian A Dement WC The sleep apnea syndromes. Annu Rev Med. (1976) 27:465–84. 10.1146/annurev.me.27.020176.002341 180875

[43] Bassetti C Aldrich MS Chervin RD Quint D. Sleep apnea in patients with transient ischemic attack and stroke: a prospective study of 59 patients. Neurology. (1996) 47:1167–73. 10.1212/WNL.47.5.1167 8909424

[44] Berry RB Budhiraja R Gottlieb DJ Gozal D Iber C Kapur VK. Rules for scoring respiratory events in sleep: update of the 2007 AASM Manual for the Scoring of Sleep and Associated Events. Deliberations of the Sleep Apnea Definitions Task Force of the American Academy of Sleep Medicine. J Clin Sleep Med. (2012) 8:597–619. 10.5664/jcsm.2172 23066376 PMC3459210

[45] Collop NA. Obstructive sleep apnea syndromes. Semin Respir Crit Care Med. (2005) 26:13–24. 10.1055/s-2005-864198 16052414

[46] Bucks RS Olaithe M Eastwood P. Neurocognitive function in obstructive sleep apnoea: a meta-review. Respirology. (2013) 18:61–70. 10.1111/j.1440-1843.2012.02255.x 22913604

[47] Yaggi HK Concato J Kernan WN Lichtman JH Brass LM Mohsenin V. Obstructive sleep apnea as a risk factor for stroke and death. N Engl J Med. (2005) 353:2034–41. 10.1056/NEJMoa043104 16282178

[48] Rosenzweig I Glasser M Polsek D Leschziner GD Williams SCR Morrell MJ. Sleep apnoea and the brain: a complex relationship. Lancet Respir Med. (2015) 3:404–14. 10.1016/S2213-2600(15)00090-9 25887982

[49] Livera LN Spencer SA Thorniley MS Wickramasinghe YA Rolfe P. Effects of hypoxaemia and bradycardia on neonatal cerebral haemodynamics. Arch Dis Child. (1991) 66:376–80. 10.1136/adc.66.4_Spec_No.376 2025027 PMC1590310

[50] Hoshi Y Mizukami S Tamura M Dynamic features of hemodynamic and metabolic changes in the human brain during all-night sleep as revealed by near-infrared spectroscopy. Brain Res. (1994) 652:257–62. 10.1016/0006-8993(94)90235-6 7953738

[51] Shiotsuka S Atsumi Y Ogata S Yamamoto R Igawa M Takahashi K. Cerebral blood volume in the sleep measured by near-infrared spectroscopy. Psychiatry Clin Neurosci. (1998) 52:172–3. 10.1111/j.1440-1819.1998.tb01012.x 9628133

[52] Kubota Y Takasu NN Horita S Kondo M Shimizu M Okada T. Dorsolateral prefrontal cortical oxygenation during REM sleep in humans. Brain Res. (2011) 1389:83–92. 10.1016/j.brainres.2011.02.061 21382356

[53] Metz AJ Pugin F Huber R Achermann P Wolf M. Changes of cerebral tissue oxygen saturation at sleep transitions in adolescents. Adv Exp Med Biol. (2014) 812:279–85. 10.1007/978-1-4939-0620-8_37 24729244

[54] Zhang Z Khatami R. A biphasic change of regional blood volume in the frontal cortex during non-rapid eye movement sleep: a near-infrared spectroscopy study. Sleep. (2015) 38:1211–7. 10.5665/sleep.4894 25761983 PMC4507726

[55] Spielman AJ Zhang G Yang CM D'Ambrosio P Serizawa S Nagata M. Intracerebral hemodynamics probed by near infrared spectroscopy in the transition between wakefulness and sleep. Brain Res. (2000) 866:313–25. 10.1016/S0006-8993(00)02320-9 10825508

[56] Zhang Z Khatami R. Predominant endothelial vasomotor activity during human sleep: a near-infrared spectroscopy study. Eur J Neurosci. (2014) 40:3396–404. 10.1111/ejn.12702 25156240

[57] Terzano MG Parrino L Sherieri A Chervin R Chokroverty S Guilleminault C. Atlas, rules, and recording techniques for the scoring of cyclic alternating pattern (CAP) in human sleep. Sleep Med. (2001) 2:537–53. 10.1016/S1389-9457(01)00149-6 14592270

[58] Näsi T Virtanen J Toppila J Salmi T Ilmoniemi RJ. Cyclic alternating pattern is associated with cerebral hemodynamic variation: a near-infrared spectroscopy study of sleep in healthy humans. PLoS ONE. (2012) 7:e46899. 10.1371/journal.pone.0046899 23071658 PMC3468598

[59] Metz AJ Pugin F Huber R Achermann P Wolf M. Brain tissue oxygen saturation increases during the night in adolescents. Adv Exp Med Biol. (2013) 789:113–9. 10.1007/978-1-4614-7411-1_16 23852484

[60] Aritake S Higuchi S Suzuki H Kuriyama K Enomoto M Soshi T. Increased cerebral blood flow in the right frontal lobe area during sleep precedes self-awakening in humans. BMC Neurosci. (2012) 13:153. 10.1186/1471-2202-13-153 23256572 PMC3538054

[61] Eiselt M Schendel M Witte H Dörschel J Curzi-Dascalova L D'Allest AM. Quantitative analysis of discontinuous EEG in premature and full-term newborns during quiet sleep. Electroencephalogr Clin Neurophysiol. (1997) 103:528–34. 10.1016/S0013-4694(97)00033-3 9402883

[62] Roche-Labarbe N Wallois F Ponchel E Kongolo G Grebe R. Coupled oxygenation oscillation measured by NIRS and intermittent cerebral activation on EEG in premature infants. Neuroimage. (2007) 36:718–27. 10.1016/j.neuroimage.2007.04.002 17482837

[63] Kato I Franco P Groswasser J Scaillet S Kelmanson I Togari H. Incomplete arousal processes in infants who were victims of sudden death. Am J Respir Crit Care Med. (2003) 168:1298–303. 10.1164/rccm.200301-134OC 12917226

[64] Zotter H Urlesberger B Kerbl R Mueller W Pichler G Curzi-Dascalova L. Cerebral hemodynamics during arousals in preterm infants. Early Hum Dev. (2007) 83:239–46. 10.1016/j.earlhumdev.2006.05.019 16828990

[65] Münger DM Bucher HU Duc G. Sleep state changes associated with cerebral blood volume changes in healthy term newborn infants. Early Hum Dev. (1998) 52:27–42. 10.1016/S0378-3782(98)00002-4 9758246

[66] Albani M Bentele KHP Budde C Schulte FJ. Infant sleep apnea profile: preterm vs. term infants. Eur J Pediatrics. (1985) 143:261–8. 10.1007/BF00442298 3987727

[67] Jenni OG Bucher HU von Siebenthal K Wolf M Keel M Duc G. Cyclical variations in cerebral blood volume during periodic breathing. Acta Paediatr. (1994) 83:1095–6. 10.1111/j.1651-2227.1994.tb12993.x 7841712

[68] Urlesberger B Pichler G Gradnitzer E Reiterer F Zobel G Müller W. Changes in cerebral blood volume and cerebral oxygenation during periodic breathing in term infants. Neuropediatrics. (2000) 31:75–81. 10.1055/s-2000-7477 10832581

[69] Decima PFF Fyfe KL Odoi A Wong FY Horne RSC. The longitudinal effects of persistent periodic breathing on cerebral oxygenation in preterm infants. Sleep Med. (2015) 16:729–35. 10.1016/j.sleep.2015.02.537 25959095

[70] Horne RSC Sun S Yiallourou SR Fyfe KL Odoi A Wong FY. Comparison of the longitudinal effects of persistent periodic breathing and apnoea on cerebral oxygenation in term- and preterm-born infants. J Physiol. (2018) 596:6021–31. 10.1113/JP275686 29528500 PMC6265532

[71] Khadra MA McConnell K VanDyke R Somers V Fenchel M Quadri S. Determinants of regional cerebral oxygenation in children with sleep-disordered breathing. Am J Respir Crit Care Med. (2008) 178:870–5. 10.1164/rccm.200802-321OC 18658114 PMC2566795

[72] Olmo Arroyo J Khirani S Amaddeo A Griffon L De Sanctis L Pouard P. A comparison of pulse oximetry and cerebral oxygenation in children with severe sleep apnea-hypopnea syndrome: a pilot study. J Sleep Res. (2017) 26:799–808. 10.1111/jsr.12561 28560835

[73] Tamanyan K Weichard A Biggs SN Davey MJ Nixon GM Walter LM. The impact of central and obstructive respiratory events on cerebral oxygenation in children with sleep disordered breathing. Sleep. (2019) 42:zsz044. 10.1093/sleep/zsz044 30958878

[74] Tamanyan K Walter LM Weichard A Davey MJ Nixon GM Biggs SN. Age effects on cerebral oxygenation and behavior in children with sleep-disordered breathing. Am J Respir Crit Care Med. (2018) 197:1468–77. 10.1164/rccm.201709-1825OC 29351000

[75] Hayakawa T Terashima M Kayukawa Y Ohta T Okada T. Changes in cerebral oxygenation and hemodynamics during obstructive sleep apneas. Chest. (1996) 109:916–21. 10.1378/chest.109.4.916 8635370

[76] Valipour A McGown AD Makker H O'Sullivan C Spiro SG. Some factors affecting cerebral tissue saturation during obstructive sleep apnoea. Eur Respir J. (2002) 20:444–50. 10.1183/09031936.02.00265702 12212980

[77] Matsuo A Inoue Y Namba K Chiba H. Changes in cerebral hemoglobin indices in obstructive sleep apnea syndrome with nasal continuous positive airway pressure treatment. Sleep Breath. (2011) 15:487–92. 10.1007/s11325-010-0367-y 20589535

[78] Zhang Z Schneider M Fritschi U Lehner I Khatami R Near-infrared spectroscopy (NIRS) as a useful tool to evaluate the treatment efficacy of positive airways pressure therapy in patients with obstructive sleep apnea syndrome (OSAS): a pilot study. J Innov Opt Health Sci. (2013) 7:1450014 10.1142/S179354581450014X

[79] Zhang Z Schneider M Laures M Qi M Khatami R. The comparisons of cerebral hemodynamics induced by obstructive sleep Apnea with Arousal and periodic limb movement with arousal: a pilot NIRS study. Front Neurosci. (2016) 10:403. 10.3389/fnins.2016.00403 27630539 PMC5005379

[80] Virtanen J Noponen T Salmi T Toppila J Meriläinen P. Impaired cerebral vasoreactivity may cause cerebral blood volume dip following obstructive sleep apnea termination. Sleep Breath. (2012) 16:309–12. 10.1007/s11325-011-0526-9 21553349

[81] Jenni OG Wolf M Hengartner M Siebenthal K Keel M Bucher HU. Impact of central, obstructive and mixed apnea on cerebral hemodynamics in preterm infants. Biol Neonate. (1996) 70:91–100. 10.1159/000244353 8864428

[82] Pizza F Biallas M Wolf M Werth E Bassetti CL. Nocturnal cerebral hemodynamics in snorers and in patients with obstructive sleep apnea: a near-infrared spectroscopy study. Sleep. (2010) 33:205–10. 10.1093/sleep/33.2.205 20175404 PMC2817907

[83] Pizza F Biallas M Kallweit U Wolf M Bassetti CL. Cerebral hemodynamic changes in stroke during sleep-disordered breathing. Stroke. (2012) 43:1951–53. 10.1161/STROKEAHA.112.656298 22693128

[84] Pizza F Biallas M Wolf M Valko PO Bassetti CL. Periodic leg movements during sleep and cerebral hemodynamic changes detected by NIRS. Clin Neurophysiol. (2009) 120:1329–34. 10.1016/j.clinph.2009.05.009 19540159

[85] Olopade CO Mensah E Gupta R Huo D Picchietti DL Gratton E. Noninvasive determination of brain tissue oxygenation during sleep in obstructive sleep apnea: a near-infrared spectroscopic approach. Sleep. (2007) 30:1747–55. 10.1093/sleep/30.12.1747 18246984 PMC2276122

[86] Payer C Urlesberger B Pauger M Müller W. Apnea associated with hypoxia in preterm infants: impact on cerebral blood volume. Brain Dev. (2003) 25:25–31. 10.1016/s0387-7604(02)00121-3 12536030

[87] Urlesberger B Kaspirek A Pichler G Müller W. Apnoea of prematurity and changes in cerebral oxygenation and cerebral blood volume. Neuropediatrics. (1999) 30:29–33. 10.1055/s-2007-973453 10222458

[88] Lagercrantz H. Improved understanding of respiratory control -implications for the treatment of apnoea. Eur J Pediatrics. (1995) 154:S10–2. 10.1007/BF02155104 7588981

[89] Safonova LP Michalos A Wolf U Choi JH Wolf M Mantulin WW Diminished cerebral circulatory autoregulation in obstructive sleep apnea investigated by near-infrared spectroscopy. Sleep Res. Online. (2003) 5:123–32.

[90] Schytz HW Jensen BE Jennum P Selb J Boas DA Ashina M. Low-frequency oscillations and vasoreactivity of cortical vessels in obstructive sleep apnea during wakefulness: a near infrared spectroscopy study. Sleep Med. (2013) 14:416–21. 10.1016/j.sleep.2012.12.009 23517585 PMC6420812

[91] Ullman N Anas NG Izaguirre E Haugen W Ortiz H Arguello O. Usefulness of cerebral NIRS in detecting the effects of pediatric sleep apnea. Pediatr Pulmonol. (2014) 49:1036–42. 10.1002/ppul.22962 24339172

[92] Schmid MB Hopfner RJ Lenhof S Hummler HD Fuchs H. Cerebral oxygenation during intermittent hypoxemia and bradycardia in preterm infants. Neonatology. (2015) 107:137–46. 10.1159/000368294 25531368

[93] Horne RSC Fung ACH NcNeil S Fyfe KL Odoi A Wong FY. The longitudinal effects of persistent apnea on cerebral oxygenation in infants born preterm. J Pediatrics. (2017) 182:79–84. 10.1016/j.jpeds.2016.11.081 28063687

[94] Watkin SL Spencer SA Dimmock PW Wickramasinghe YA Rolfe P. A comparison of pulse oximetry and near infrared spectroscopy (NIRS) in the detection of hypoxaemia occurring with pauses in nasal airflow in neonates. J Clin Monit Comput. (1999) 15:441–7. 10.1023/A:1009938225495 12578041

[95] Yamamoto A Yokoyama N Yonetani M Uetani Y Nakamura H Nakao H. Evaluation of change of cerebral circulation by SpO_2_ in preterm infants with apneic episodes using near infrared spectroscopy. Pediatr Int. (2003) 45:661–4. 10.1111/j.1442-200X.2003.01803.x 14651537

[96] Rupp T Peyrard A Tamisier R Pepin J-L Verges S. Cerebral and muscle oxygenation during intermittent hypoxia exposure in healthy humans. Sleep. (2016) 39:1197–9. 10.5665/sleep.5830 26951398 PMC4863206

[97] Schwarz EI Furian M Schlatzer C Stradling JR Kohler M Bloch KE. Nocturnal cerebral hypoxia in obstructive sleep apnoea: a randomised controlled trial. Eur Respir J. (2018) 51:1800032. 10.1183/13993003.00032-2018 29700104

[98] Uchida-Ota M Tanaka N Sato H Maki A. Intrinsic correlations of electroencephalography rhythms with cerebral hemodynamics during sleep transitions. Neuroimage. (2008) 42:357–68. 10.1016/j.neuroimage.2008.03.055 18514543

[99] Fultz NE Bonmassar G Setsompop K Stickgold RA Rosen BR Polimeni JR. Coupled electrophysiological, hemodynamic, and cerebrospinal fluid oscillations in human sleep. Science. (2019) 366:628–31. 10.1126/science.aax5440 31672896 PMC7309589

[100] Walter LM Tamanyan K Weichard AJ Davey MJ Nixon GM Horne RSC. Sleep disordered breathing in children disrupts the maturation of autonomic control of heart rate and its association with cerebral oxygenation. J Physiol. (2019) 597:819–30. 10.1113/JP276933 30471111 PMC6355722

[101] Kotajima F Meadows GE Morrell MJ Corfield DR. Cerebral blood flow changes associated with fluctuations in alpha and theta rhythm during sleep onset in humans. J Physiol. (2005) 568:305–13. 10.1113/jphysiol.2005.092577 16002438 PMC1474761

[102] Obrig H Wolf T Döge C Hülsing JJ Dirnagl U Villringer A. Cerebral oxygenation changes during motor and somatosensory stimulation in humans, as measured by near-infrared spectroscopy. Adv Exp Med Biol. (1996) 388:219–24. 10.1007/978-1-4613-0333-6_27 8798815

[103] Obrig H Hirth C Junge-Hülsing JG Döge C Wolf T Dirnagl U. Cerebral oxygenation changes in response to motor stimulation. J Appl Physiol. (1996) 81:1174–83. 10.1152/jappl.1996.81.3.1174 8889751

[104] Hoshi Y Kobayashi N Tamura M. Interpretation of near-infrared spectroscopy signals: a study with a newly developed perfused rat brain model. J Appl Physiol. (2001) 90:1657–62. 10.1152/jappl.2001.90.5.1657 11299252

[105] Seiyama A Seki J Tanabe HC Sase I Takatsuki A Miyauchi S. Circulatory basis of fMRI signals: relationship between changes in the hemodynamic parameters and BOLD signal intensity. Neuroimage. (2004) 21:1204–14. 10.1016/j.neuroimage.2003.12.002 15050548

[106] Vyazovskiy VV Harris KD. Sleep and the single neuron: the role of global slow oscillations in individual cell rest. Nat Rev Neurosci. (2013) 14:443–51. 10.1038/nrn3494 23635871 PMC3972489

[107] Firbank M Okada E Delpy DT. A theoretical study of the signal contribution of regions of the adult head to near-infrared spectroscopy studies of visual evoked responses. Neuroimage. (1998) 8:69–78. 10.1006/nimg.1998.0348 9698577

[108] Urlesberger B Trip K Ruchti JJ Kerbl R Reiterer F Müller W. Quantification of cyclical fluctuations in cerebral blood volume in healthy infants. Neuropediatrics. (1998) 29:208–11. 10.1055/s-2007-973562 9762697

[109] Derdeyn CP Videen TO Yundt KD Fritsch SM Carpenter DA Grubb RL. Variability of cerebral blood volume and oxygen extraction: stages of cerebral haemodynamic impairment revisited. Brain. (2002) 125:595–607. 10.1093/brain/awf047 11872616

[110] Aalkjaer C Boedtkjer D Matchkov V. Vasomotion - what is currently thought?: Vasomotion. Acta Physiol. (2011) 202:253–69. 10.1111/j.1748-1716.2011.02320.x 21518271

[111] Rivadulla C de Labra C Grieve KL Cudeiro J. Vasomotion and neurovascular coupling in the visual thalamus *in vivo*. PLoS ONE. (2011) 6:e28746. 10.1371/journal.pone.0028746 22174886 PMC3235153

[112] Vantanajal JS Ashmead JC Anderson TJ Hepple RT Poulin MJ. Differential sensitivities of cerebral and brachial blood flow to hypercapnia in humans. J Appl Physiol. (2007) 102:87–93. 10.1152/japplphysiol.00772.2006 17023571

[113] Antonelli Incalzi R Marra C Salvigni BL Petrone A Gemma A Selvaggio D. Does cognitive dysfunction conform to a distinctive pattern in obstructive sleep apnea syndrome? J Sleep Res. (2004) 13:79–86. 10.1111/j.1365-2869.2004.00389.x 14996039

[114] Kohler M Stradling JR. Mechanisms of vascular damage in obstructive sleep apnea. Nat Rev Cardiol. (2010) 7:677–85. 10.1038/nrcardio.2010.145 21079639

[115] Carlson JT Hedner J Elam M Ejnell H Sellgren J Wallin BG. Augmented resting sympathetic activity in awake patients with obstructive sleep apnea. Chest. (1993) 103:1763–8. 10.1378/chest.103.6.1763 8404098

[116] Tachtsidis I Elwell CE Leung TS Lee C-W Smith M Delpy DT. Investigation of cerebral haemodynamics by near-infrared spectroscopy in young healthy volunteers reveals posture-dependent spontaneous oscillations. Physiol Meas. (2004) 25:437–45. 10.1088/0967-3334/25/2/003 15132309

[117] Bixler EO Vgontzas AN Ten Have T Tyson K Kales A. Effects of age on sleep apnea in men: I. Prevalence and severity. Am J Respir Crit Care Med. (1998) 157:144–8. 10.1164/ajrccm.157.1.9706079 9445292

[118] Gries RE Brooks LJ. Normal oxyhemoglobin saturation during sleep. How low does it go? Chest. (1996) 110:1489–92. 10.1378/chest.110.6.1489 8989066

[119] Philip P Dealberto MJ Dartigues JF Guilleminault C Bioulac B. Prevalence and correlates of nocturnal desaturations in a sample of elderly people. J Sleep Res. (1997) 6:264–71. 10.1111/j.1365-2869.1997.00264.x 9493527

[120] Leenders KL Perani D Lammertsma AA Heather JD Buckingham P Healy MJ. Cerebral blood flow, blood volume and oxygen utilization. Normal values and effect of age. Brain. (1990) 113(Pt 1):27–47. 10.1093/brain/113.1.27 2302536

[121] Naritomi H Meyer JS Sakai F Yamaguchi F Shaw T. Effects of advancing age on regional cerebral blood flow: studies in normal subjects and subjects with risk factors for atherothrombotic stroke. Arch Neurol. (1979) 36:410–6. 10.1001/archneur.1979.00500430040005 454246

[122] Meyer JS Rauch G Rauch RA Haque A. Risk factors for cerebral hypoperfusion, mild cognitive impairment, and dementia. Neurobiol Aging. (2000) 21:161–9. 10.1016/S0197-4580(00)00136-6 10867201

[123] Sevick EM Chance B Leigh J Nioka S Maris M. Quantitation of time- and frequency-resolved optical spectra for the determination of tissue oxygenation. Anal Biochem. (1991) 195:330–51. 10.1016/0003-2697(91)90339-U 1750689

[124] Peigneux P. Neuroimaging studies of sleep and memory in humans. Curr Top Behav Neurosci. (2015) 25:239–68. 10.1007/7854_2014_326 24961345

[125] Miller EK Freedman DJ Wallis JD. The prefrontal cortex: categories, concepts and cognition. Philos Trans R Soc Lond B Biol Sci. (2002) 357:1123–36. 10.1098/rstb.2002.1099 12217179 PMC1693009

[126] Muzur A Pace-Schott EF Hobson JA. The prefrontal cortex in sleep. Trends Cogn Sci. (2002) 6:475–81. 10.1016/S1364-6613(02)01992-7 12457899

[127] Broughton R Hasan J. Quantitative topographic electroencephalographic mapping during drowsiness and sleep onset. J Clin Neurophysiol. (1995) 12:372–86. 10.1097/00004691-199512040-00007 7560024

[128] Meck WH. Frontal cortex lesions eliminate the clock speed effect of dopaminergic drugs on interval timing. Brain Res. (2006) 1108:157–67. 10.1016/j.brainres.2006.06.046 16844101

[129] Saager RB Telleri NL Berger AJ. Two-detector Corrected Near Infrared Spectroscopy (C-NIRS) detects hemodynamic activation responses more robustly than single-detector NIRS. Neuroimage. (2011) 55:1679–85. 10.1016/j.neuroimage.2011.01.043 21256223

[130] Khaksari K Blaney G Sassaroli A Krishnamurthy N Pham T Fantini S. Depth dependence of coherent hemodynamics in the human head. JBO. (2018) 23:121615. 10.1117/1.JBO.23.12.121615 30444084 PMC6318717

[131] Brigadoi S Ceccherini L Cutini S Scarpa F Scatturin P Selb J. Motion artifacts in functional near-infrared spectroscopy: a comparison of motion correction techniques applied to real cognitive data. Neuroimage. (2014) 85(Pt 1):181–91. 10.1016/j.neuroimage.2013.04.082 23639260 PMC3762942

[132] Cooper RJ Selb J Gagnon L Phillip D Schytz HW Iversen HK. A systematic comparison of motion artifact correction techniques for functional near-infrared spectroscopy. Front Neurosci. (2012) 6:147. 10.3389/fnins.2012.00147 23087603 PMC3468891

[133] Kooi EMW Verhagen EA Elting JWJ Czosnyka M Austin T Wong FY. Measuring cerebrovascular autoregulation in preterm infants using near-infrared spectroscopy: an overview of the literature. Expert Rev Neurotherap. (2017) 17:801–18. 10.1080/14737175.2017.1346472 28639837

[134] Tau GZ Peterson BS. Normal development of brain circuits. Neuropsychopharmacology. (2010) 35:147–68. 10.1038/npp.2009.115 19794405 PMC3055433

[135] Biessmann F Plis S Meinecke FC Eichele T Muller K-R. Analysis of multimodal neuroimaging data. IEEE Rev Biomed Eng. (2011) 4:26–58. 10.1109/RBME.2011.2170675 22273790

[136] Jiang X Gu X Xu K Ren H Chen W Independent decision path fusion for bimodal asynchronous brain-computer interface to discriminate multiclass mental states. IEEE Access. (2019) 7:1 10.1109/ACCESS.2019.2953535

